# Cross-sectional stratification of fall-related vulnerability in Parkinson’s disease through multimodal fusion of speech, gait, facial expression, and upper-limb movement

**DOI:** 10.3389/fdgth.2026.1937343

**Published:** 2026-09-23

**Authors:** Vasileios Skaramagkas, Iro Boura, Georgios Karamanis, Anastasia Pentari, Emilia Michou, Konstantinos Marias, Dimitrios I. Fotiadis, Zinovia Kefalopoulou, Cleanthe Spanaki, Manolis Tsiknakis

**Affiliations:** 1 Electrical and Computer Engineering Department, Hellenic Mediterranean University, Heraklion, Greece; 2 Institute of Computer Science, Foundation for Research and Technology Hellas (FORTH), Heraklion, Greece; 3 ARTORG Centre for Biomedical Engineering Research, University of Bern, Bern, Switzerland; 4 School of Medicine, University of Crete, Heraklion, Greece; 5 Department of Neurology, University Hospital of Heraklion, Heraklion, Greece; 6 School of Medicine, University of Patras, Patras, Greece; 7 Department of Neurology, Patras University Hospital, Patras, Greece; 8 Department of Speech and Language Therapy, School of Health Rehabilitation Sciences, University of Patras, Patras, Greece; 9 Unit of Medical Technology and Intelligent Information Systems, University of Ioannina, Ioannina, Greece; 10 Biomedical Research Institute, Foundation for Research and Technology Hellas (FORTH), Ioannina, Greece

**Keywords:** digital biomarkers, fall-related vulnerability stratification, model pruning, multimodal deep learning, Parkinson’s disease

## Abstract

Falls constitute a significant cause of morbidity in Parkinson’s disease (PD), but current approaches to cross-sectional assessment of fall-related vulnerability frequently depend on unimodal mobility metrics with limited ability to characterize its multidimensional manifestations. This study presents a multimodal deep learning (DL) architecture that incorporates facial expressions, voice, gait dynamics, and upper-limb movements to classify cross-sectional fall-related vulnerability in PD using current postural-instability status and fall history. Data were gathered from 147 patients, and modality-specific models were systematically pruned to reduce model size, inference latency, and computational requirements while maintaining classification performance, providing an initial assessment of computational efficiency within the experimental environment. Various fusion strategies, including late, intermediate, attention-based, transformer-based, and large-language-model-assisted methods, were assessed using leave-one-subject-out cross-validation. The large-language-model-assisted analysis was exploratory and was based on a single recorded API run per participant–modality configuration. Pruning resulted in considerable efficiency improvements without a notable decrease in accuracy, with gait demonstrating the highest robustness. Fusion performance varied across methods and modality combinations. Attention-based fusion with face and gait achieved an observed macro-f1 of 91.67%. An exploratory large-language-model-assisted face–upper-limb configuration produced an observed macro-f1 of 93.75% in a single recorded run; because repeated-run stability and exact model-version reproducibility were not established, this result should not be interpreted as evidence of reproducible superiority. Transformer fusion produced an observed macro-f1 of 85.02% when all four modalities were included. Significantly, speech and particularly face expressivity provided supplementary signals to conventional gait and upper-limb assessments, underscoring the potential of underexplored axial characteristics as complementary digital markers associated with fall-related vulnerability. These findings support further study of multimodal AI for cross-sectional classification of fall-related vulnerability in PD. Prospective fall prediction, external generalizability, and performance on intended devices remain to be evaluated in independent future studies.

## 1 Introduction

Parkinson’s disease (PD) is the second most common neurodegenerative disease, anticipated to impact almost 15.6 million individuals worldwide by 2030 (1). Large population-based studies indicate that late-stage PD (≥10 years) and its terminal phase are associated with the greatest disability and economic burden (2, 3). These costs, which are expected to rise further, are particularly high among older individuals (≥65 years) (3), and are largely driven by hospitals, nursing homes and home care (2), with a single hospitalization often triggering repeated admissions (4). Despite therapeutic advances, PD inevitably progresses over time, leading to accumulating limitations in daily function; slowing or preventing disability remains, therefore, a central goal of clinical management (5).

Falls are common among PD patients, with up to 90% experiencing at least one, and nearly 40% reporting recurrent falls (6). They are multifactorial in nature, occurring as a result of disease progression, aging, comorbidities, polypharmacy, including antidepressants or higher dopaminergic doses, vitamin B12 deficiency, and specific non-motor symptoms such as dysautonomia, cognitive impairment, psychosis, and sleep problems (7–9). Falls are a major contributor to hospitalizations, poor quality of life, reduced mobility, and increased mortality in PD (9). Because disability strongly predicts falls, and falls per se drive morbidity and institutional care, targeted strategies to mitigate fall risk could substantially improve outcomes while easing healthcare costs (10–12). Effective fall-prevention measures include patient education, structured exercise programs, and physical or occupational therapy, including remote delivery of such services, as well as the use of appropriate footwear or assistive devices (13). Nevertheless, engagement in such interventions is often hindered by fear of falling, non-motor symptoms (e.g., fatigue, anxiety, pain, apathy), and limited access to specialized services (14). Multimodal technologies may provide additional information for cross-sectional assessment; whether such technologies can prospectively identify future fallers or guide the timing of interventions requires separate longitudinal evaluation.

Conventional risk assessment techniques for fall estimation in PD mostly rely on mobility measurements, especially gait, alongside clinical scales and questionnaires (15, 16). Falls have been consistently linked to axial motor parkinsonian features overall, primarily those affecting gait and balance, such as postural instability, camptocormia, or freezing of gait (9, 17). However, there are a few reports that have associated falls with other axial features, including dysphagia or speech impairment (18, 19). Although machine learning models have successfully classified PD symptoms through individual modalities (e.g., force-sensor gait analysis, speech samples, facial expression videos, hand movements), there is a growing recognition that the integration of multiple sensor-based modalities may yield a more comprehensive assessment framework (20).

Recent research in PD has started integrating many modalities for diagnostic purposes, including the combination of gait and clinical metrics (21), as well as the application of audio-visual deep learning (DL) frameworks for the monitoring of motor symptoms (22). Nonetheless, multimodal classification of fall-related vulnerability, based on clinically observable balance impairment and recent fall history, has received limited attention in comprehensive DL research.

Our previous studies developed four modality-specific architectures for distinct PD-related classification tasks: (1) facial-video analysis for ON/OFF medication-state classification (23), (2) speech analysis for distinguishing PD patients from healthy controls (24), (3) pressure-insole gait analysis for differentiating treatment-related gait patterns (25), and (4) wrist-accelerometer analysis for distinguishing PD patients with minor symptoms from healthy controls (26). Thus, these earlier studies differed from the present work in both their clinical endpoints and their unimodal design. The current study does not propose these four architectures as new models. Instead, it repurposes and task-specifically retrains them on a common cohort using a three-class cross-sectional endpoint representing different levels of fall-related vulnerability, defined by current postural-instability status and fall history during the preceding six months. Its novelty lies in their patient-level harmonization within a single multimodal framework, the modality-specific compression of the constituent networks, the systematic comparison of five fusion paradigms, and the evaluation of the incremental contribution of each modality through ablation analysis. Our research therefore presents three primary contributions:

- **Comprehensive multimodal fusion for a cross-sectional clinical endpoint.** In contrast to the earlier unimodal studies, which addressed medication state, PD diagnosis, or treatment-related motor-pattern classification, the present work investigates three-class cross-sectional classification of fall-related vulnerability. We systematically compare classical late (LF) and intermediate fusion (IF), attention-based fusion (ABF), multimodal transformer fusion (MTF), and Large Language Model-assisted fusion (LLMF) while preserving modality-specific representations and enabling cross-modal interactions.
- **Modality-specific compression and computational-efficiency evaluation.** We implement structured pruning separately for each previously developed architecture and evaluate the resulting trade-offs in f1-score, model size, inference time, and computational cost. The selected compressed models are subsequently used within the fusion experiments, providing a comparative assessment of model size, inference latency, computational cost, and classification performance that was not undertaken in the original unimodal publications
- **Clinical focus on axial dysfunction.** Although gait and balance impairments are associated with falls and prospective fall risk, the discriminative contribution of speech and facial expressivity to cross-sectional classification of fall-related vulnerability remains understudied. Both hypomimia and dysarthria or hypophonia indicate midline axial dysfunction in PD, which is mechanistically linked to postural instability (27, 28); they may also reflect emotional and social behaviors, thereby capturing aspects of non-motor symptoms such as cognitive or mood disorders (29, 30). By integrating these modalities, we explore their potential as supplementary digital biomarkers that may provide information beyond mobility sensors alone.

This study therefore addresses three primary objectives:

1. To develop and compare multiple fusion architectures for three-class cross-sectional classification of fall-related vulnerability, defined by current postural instability and fall history during the preceding six months (Falls > Balance impairment/No falls > No balance impairment/No falls).
2. To implement and evaluate modality-specific pruning methods and quantify their effects on classification performance, model size, inference latency, and computational cost.
3. To conduct an ablation study that quantifies the contribution of each modality and their combinations, with a particular focus on determining whether face and speech signals provide additional discriminative information beyond those captured from gait and upper-limb data.

Thus, the primary advancement of our research lies not in the creation of the four unimodal frameworks, but in their task-specific modification and integration for cross-sectional classification of fall-related vulnerability in PD. The framework integrates traditional movement-based signals with facial and vocal data, directly assessing whether these less frequently examined modalities offer additional discriminative value beyond gait and upper-limb metrics. Together with participant-level leave-one-subject-out cross-validation, structured pruning, comparison of five fusion strategies, and systematic modality ablation, this provides a methodological contribution distinct from the aims and analyses of the four preceding papers.

## 2 Methods

### 2.1 Dataset

Participants were recruited over one year (2023–2024) from the Movement Disorders Outpatient Clinics of two hospitals in Greece, the University General Hospital of Heraklion in Crete (UGHH), and the University General Hospital of Patra (UGHP). Each participant underwent two assessments with the MDS-UPDRS-III, one after a 12-hr withdrawal of dopaminergic medications (OFF-medication or overnight OFF) and the second after resuming their dopaminergic therapy (ON-medication). They completed a series of motor, facial and vocal tasks, while wearing sensors and being video-recorded in both states. Recruitment processes, inclusion/exclusion criteria, and research settings/procedures have been described in detail elsewhere (31). For the purposes of this study, only individuals assessed in the ON-medication state and successfully completing all multimodal experimental tasks, thus, yielding data for all four modalities, were included in our final analysis. Of the 183 participants initially assessed, 147 were included in the final analysis and 36 were excluded because complete, successfully processable data were unavailable for one or more modalities. Recordings that were missing or could not be processed successfully were treated as unavailable; missing modalities were not imputed. Class imbalance was addressed in the performance evaluation by reporting macro-averaged metrics, which assign equal importance to each class. No additional class rebalancing through oversampling, undersampling, or class-weighted loss functions was applied.

Participants were later classified into three groups based on the existence of postural instability in the ON-medication state (MDS-UPDRS item 3.12=0 vs. ≥1) and the incidence of falls in the preceding six months (NMSQ item 21 = No vs. Yes). class A comprised patients exhibiting no objectively demonstrable balance deficits and lacking a history of falls. class B included patients exhibiting even slight postural instability on the pull-back test, without reporting any falls. class C consisted of those who had encountered at least one fall in the preceding six months, regardless of balance issues. This classification was designed to represent cross-sectional differences in fall-related vulnerability among ambulatory participants with PD (H&Y ≤3), many of whom remained largely functional and independent in everyday activities. The three groups were defined exclusively from postural-instability status measured during the current ON-medication assessment and self-reported fall history during the preceding six months. The intermediate group comprised participants with postural instability but no reported falls during that period. It was included as a clinically relevant cross-sectional subgroup and should not be interpreted as a prospectively identified pre-fall stage or as a group shown by this study to benefit from early or targeted fall-prevention interventions.

Comparative analyses of classes A, B, and C were performed to evaluate potential clinical disparities. The Shapiro–Wilk test was utilized on continuous variables to assess normality. Normally distributed variables were compared using one-way ANOVA, while non-normally distributed variables were evaluated with the Kruskal–Wallis test, based on their distributional qualities. The chi-square test was employed to assess binary variables.

All procedures involving human participants were conducted in accordance with the ethical standards of the institutional and national research committees and with the 1964 Helsinki Declaration and its later amendments. Written informed consent was obtained from all participants before participation in the study. The study was approved by the Ethics Committees of both hospitals. At the UGHH, approval was granted under reference number 11692/19-05-2023 by the Scientific Council chaired by George Halkiadakis, with vice-chair Stylianos Kteniadakis, and members I. Velegraki, A. Patriniakos, K. Tziovaras, and G. Sourvinos. At the UGHP, approval was granted under reference number 347/13-07-2023 by the Scientific Council chaired by Petros Maragos, with members I. Michou, A. Tsiata, S. Asimakopoulos, A. Sarantopoulos, and N. Darioti. Additionally, the analytical cohort comprised 147 statistically independent participants. Each participant contributed multiple within-participant observations, including facial-video segments, speech recordings, gait cycles, and wrist-accelerometer windows. These repeated observations increased the amount of signal available for representation learning but were not treated as independent samples for data partitioning, model selection, or performance estimation. All observations from a given participant were assigned exclusively to either the training set or the held-out test set in each leave-one-subject-out cross-validation fold. Thus, the effective sample size for performance evaluation remained 147 participants.

### 2.2 Experimental process

Facial expression recordings were performed under standardized ON- and OFF-medication states, adhering to a medication washout technique outlined in Roßkopf et al. (32). Patients were documented in a controlled setting with a 4K-resolution camera (GoPro Hero9) at 30 fps, under uniform lighting conditions (450 lux). Each subject executed six diadochokinetic facial motor tasks devoid of emotional content, chosen to exhibit indicators of hypomimia. These tasks included mouth opening/closing, still face, smile-and-kiss, and tongue movements in various directions. All video data were recorded during two distinct phases (ON and OFF medication), with the exception of drug-naïve and advanced PD patients, for whom only one phase was utilized due to clinical limitations. For a comprehensive account of the experimental and data collection methodology, comprehensive task design, justification, and configuration, please consult our prior publication. In this work, we utilized data only from the opening/closing mouth task, based on the model’s superior performance presented in the aforementioned publication (23).

Speech data were captured at 44.1 kHz in a controlled environment with high-fidelity microphone (Logitech Snowball) (33). The recording methodology comprised a sequence of standardized tests often employed to evaluate dysarthria and hypophonia in PD. Participants engaged in sustained phonation, phrase repetition, syllable repetition at diverse tempos, brief reading passages, rapid counting, voluntary coughing, and breathing tasks. Following the results of our publication (24), in this study, we used sustained phonation signals from our dataset (extended /a/, /s/, /z/).

Gait assessments were performed with pressure-sensor-integrated insoles (Moticon SCIENCE (34)), recording ground response forces and inertial data throughout standardized walking tasks. All participants executed the Walk Straight and Turn (WST) and the modified Timed Up and Go (mTUG) assessments under real-world medication conditions. Patients with PD were evaluated in both their ON and OFF medication states. The procedure was created to represent daily gait patterns and document treatment-specific motor characteristics. The data collection and preprocessing specifics, encompassing insole specifications, sensor sampling at 100 Hz, and testing processes, adhered to the protocol outlined in Chatzaki et al. (31). Dual-camera recordings supplemented insole data for visual gait validation. A comprehensive account of the experimental and data collection methodology can be found in Skaramagkas et al. (25). In this study, we used data consisting of pressure signals (1s gait cycles) recorded during WST and mTUG tests.

During the conduction of the walking tasks, the Empatica EmpracePlus wristband was used to monitor, record, and collect acceleration data (100 Hz) on the upper limb movement of patients, fixed to the patient’s “weak” hand’s wrist (35). Data collection from the pressure insoles and the wristband was synchronized to a precision of one-hundredth of a second. Details on the handling of the acceleration data can be found in Skaramagkas et al. (26).

### 2.3 Models and modalities

This research amalgamates four complementary sensor modalities—facial video, speech, upper limb movement, and foot pressure—each analyzed using previously established DL architectures developed for different PD-related clinical objectives. Our objective is to re-tune and integrate these systems into a unified classification framework, assessing their individual contributions and potential complementarity. To enable clinically meaningful analysis, the cohort was stratified into three classes reflecting progressively impaired balance profiles: Class A (no postural instability and no reported falls), Class B (postural instability without reported falls), and Class C (at least one reported fall during the preceding six months, irrespective of postural instability). Table 1 summarizes the core design, input format and original task for each model whilst Figure 1 demonstrates the architecture of each model.

**Table 1:** Summary of modality-specific models used for fusion.

Modality	Model architecture	Input type	Original task
Face (23)	Dual-stream Transformer (ResNet-50 + Optical Flow + Transformer Encoder)	1-s video segments	Medication state classification (ON/OFF)
Speech (24)	Hybrid model (MFCCs + RQA + VGG16 on Mel-spectrograms) + SVM/RF	Sustained vowel phonemes (/a/, /e/, /i/, /o/, /u/)	PD vs. control classification
Gait (25)	Dual-stream Vision Transformer (recurrence plots of heel/toe pressure)	1-s normalized pressure cycles	DBS vs. non-DBS vs. control gait pattern classification
Upper Limb (26)	Bi-LSTM + Attention	Handcrafted features from 1-s wrist sensor windows	PD vs. control classification

**Figure 1:**
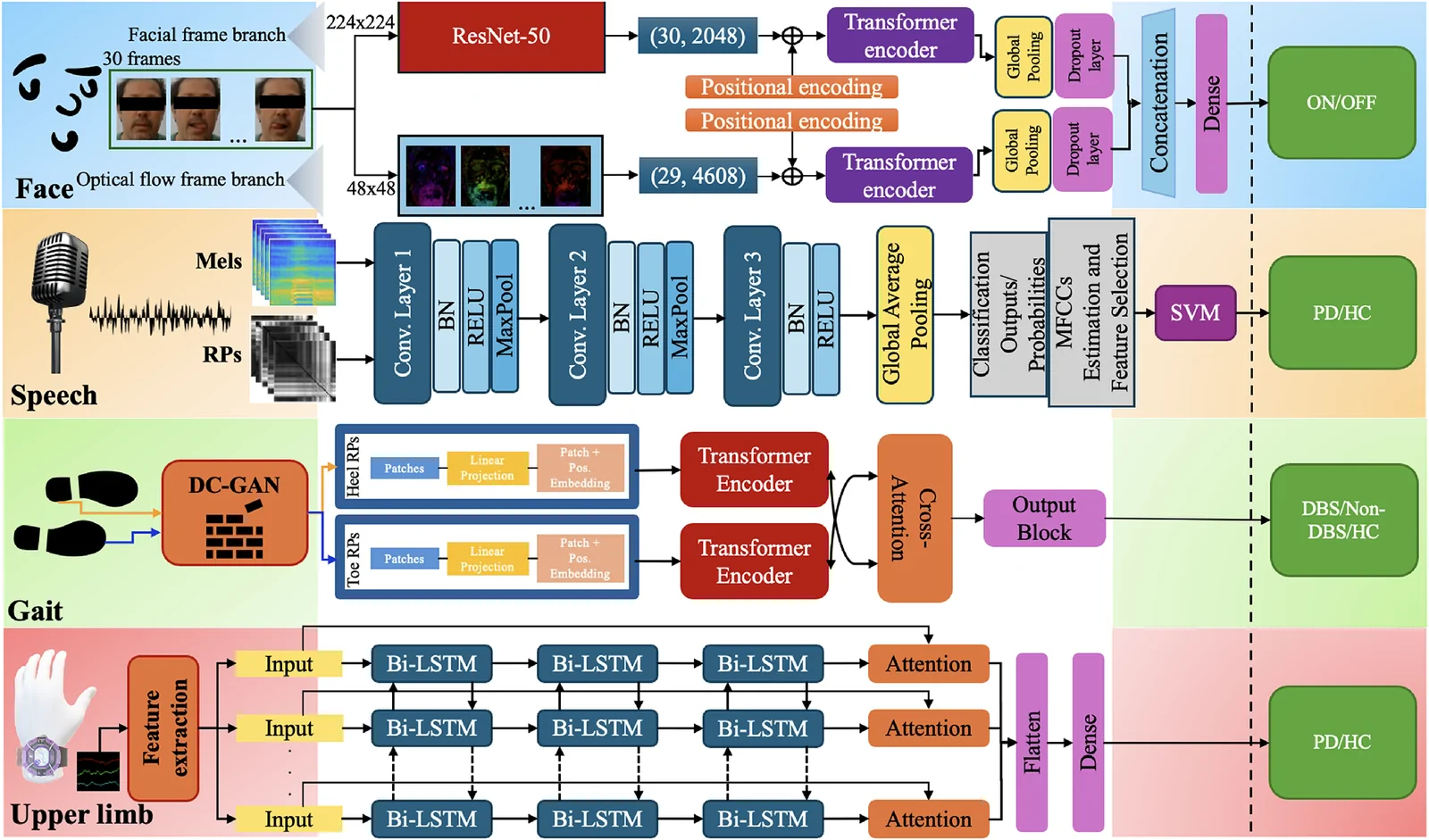
The four complimentary sensor modalities, facial video, speech, upper limb movement, and foot pressure, each analyzed using previously established deep learning architectures designed for distinct clinical objectives related to PD.

The facial video model employs a dual-stream architecture combining appearance-based features from ResNet-50 with motion dynamics captured via dense optical flow, both processed through Transformer encoders. This architecture achieved 86% accuracy in classifying ON/OFF medication states across PD patients (23).

The speech model takes a hybrid approach, combining time-frequency (MFCCs), nonlinear (RQA), and deep (VGG16 Mel-spectrogram) features, which are fused and classified via SVM or Random Forests. Among sustained vowels from PC-GITA database (36), the /a/ phoneme achieved the highest classification accuracy 96.2% (24).

The gait model processes recurrence plots derived from heel and toe pressure data using a dual-stream Vision Transformer; this approach obtained over 94% accuracy in multiclass tasks and over 98% in DBS vs non-DBS binary classification (25).

Finally, the upper limb model is a Bi-LSTM network enhanced with an attention mechanism, trained on 33 handcrafted features from triaxial wrist acceleration data (37). It reached an average accuracy 98% in distinguishing PD patients from healthy controls in naturalistic settings (26). All models were evaluated using rigorous Leave-One-Subject-Out Cross-Validation (LOSOCV or stratified folds), and their strong standalone performance supports their integration into a multi-modal framework to address a unified clinical question.

### 2.4 Pruning approach

We implemented modality-specific structured pruning methods across all DL models to improve computational efficiency while maintaining classification performance. Pruning was performed retrospectively on fully trained networks, concentrating on the elimination of entire neurons or output channels along the output dimension. This method provides structured, hardware-relevant compression by removing complete neurons or output channels (38).

The Dual Stream Transformer face model was pruned using global structured L1-norm pruning. Targets included the projection weights matrices of multi-head self-attention layers and all linear transformations. A fixed pruning ratio was applied uniformly across layers. Meanwhile, a structured pruning sweep was applied to a custom CNN used for feature extraction from Mel-spectrogram and recurrence plot images at the speech model. Each convolutional layer was pruned by reducing its output channels, maintaining a minimum number per layer to ensure representational capacity. The resulting features were fused with MFCC and RQA descriptors and classified via an SVM. The dual-stream Vision Transformer gait model underwent structured L2-norm pruning across all linear layers within the transformer blocks, fusion MLP, and classification head. Compression was applied globally to output neurons over the same pruning ratio range. Additionally, for the Bi-LSTM architecture with attention of the upper limb model, structured L1-norm pruning was applied to linear layers, LSTM weights, and the learnable attention weight matrix.

Pruning was performed at various sparsity levels, ranging from 10 to 90%. Subsequent to pruning, all models underwent fine-tuning on their designated datasets to recover any loss in classification performance. Fine-tuning entailed supervised training for 30 epochs utilizing either binary or categorical cross-entropy losses alongside suitable optimizers (Adam or AdamW). Evaluation was performed with LOSOCV, and performance was measured by conventional measures, encompassing accuracy, precision, recall, f1-score, inference time, model size, and effective sparsity. All pruning reparameterizations were removed before model saving so that the reported model files reflected the final compressed architectures.

### 2.5 Fusion strategy

We employed five fusion algorithms to combine information across the four data modalities, face, speech, gait, and upper limb: IF, LF, ABF, MTF, and LLMF. Each technique was evaluated for its ability to combine complementary information for cross-sectional classification of the three prespecified fall-related vulnerability groups (39).

IF entailed extracting latent representations from each modality-specific model and merging them into a cohesive feature vector for each patient. In the facial modality, representations from both frame-based and optical-flow-based transformer branches were aggregated and combined. Meanwhile, speech data were condensed to the top-k features determined by the Kruskal-Wallis test and averaged across samples. The resultant fused vector was employed for final classification through a fully connected neural head. This method facilitated joint learning inside the common representation space. Gait features were derived from the final transformer tokens of a dual-stream Vision Transformer, while upper limb embeddings were obtained from the attention-weighted outputs of a Bi-LSTM model.

LF functioned at the decision level. Each unimodal model generated class probability distributions by softmax. The fusion was accomplished by various rule-based methodologies: unweighted averaging, sample-count-based weighted averaging, maximum-confidence weighting, product of experts, and logit summation. Additionally, majority voting was applied to predicted class labels. This method maintains modality-specific predictions while facilitating adaptable ensemble decision-making.

ABF utilized a learnable attention mechanism to assign modality-specific weights to intermediate features prior to final classification. The embedding of each modality was processed through a gated attention block to derive a significance score, and the weighted aggregate of all features was fed into a common classifier. This enables the model to flexibly adjust its reliance on several modalities based on the quality of patient-specific signals and the contribution of each modality.

MTF utilized a transformer encoder architecture to model inter-modality dependencies. Following the projection of all modality-specific embeddings into a unified latent space, a [CLS] token (classify token) was added and positionally encoded. A series of transformer layers subsequently processed the sequence, facilitating self-attention across the modalities. The output from the [CLS] token was processed through a classification head to generate the final estimation. This architecture adeptly encapsulats global interactions across modalities while maintaining resilience to absent or distorted inputs.

LLMF was evaluated as an exploratory decision-level fusion approach using the OpenAI Chat Completions API. The API request specified the model alias gpt-5. For each held-out participant and modality combination, the user prompt contained the participant identifier and, for every available modality, the predicted probabilities for classes A, B, and C, the number of contributing observations, and the maximum predicted probability as a confidence indicator. The implementation allowed optional modality-reliability priors; however, no such priors were supplied in the reported experiments. The ground-truth class was retrieved only after the LLM response had been generated and was not included in the prompt.

The exact system message was:

You are a careful clinical decision assistant.

Return compact JSON only.

The user-message template was:

Patient ID: [patient identifier]

Task: Determine the most likely class (A, B, or C) for this PD patient, based on multimodal model outputs. Use your own reasoning on which signals to trust (confidence, sample count, and prior reliability).

Modality reliability priors (val f1 or accuracy): [optional modality-specific priors; not supplied in the reported analyses]

Modalities:

- [modality]58; probs A=[pA], B=[pB], C=[pC];

n=[number of observations];

max_conf=[maximum predicted probability]

Output JSON with keys:

predicted_label: A, B, or C;

rationale: no more than 30 words;

modal_weights: optional mapping from modality names to values between 0 and 1 reflecting relative trust.

Return no additional text.

The request specified JSON-object response formatting. Temperature, top-p, maximum output length, frequency penalty, presence penalty, and an API-level random seed were not explicitly set; therefore, the provider defaults active at the time of execution were used. Each participant–modality configuration was queried once, and repeated-run agreement was not evaluated.

The returned message content was parsed using a JSON parser. If direct JSON parsing failed, the implementation attempted to recover the substring between the first opening and last closing brace and parse that substring as JSON. The field predicted_label was then extracted and accepted only if it was one of A, B, or C; otherwise, the run was treated as invalid and raised an error. The optional rationale and modal_weights fields were also retained when present. A total of 1,617 API calls were attempted across the 11 modality combinations (147 participants × 11 combinations). All 1,617 calls completed successfully and returned a valid class label from {A, B, C}. No API calls failed, produced invalid output, or were skipped. Consequently, every LLMF performance estimate was calculated using all 147 participants. Because the analysis used a mutable model alias, unspecified generation parameters, and a single query per configuration, LLMF was treated as an exploratory proof-of-concept rather than as a reproducible benchmark or validated clinical strategy.

All fusion strategies were evaluated using LOSOCV at the patient level. Performance was compared using metrics including accuracy, precision, recall, and f1-score. For every LOSO fold, all observations belonging to the held-out participant were excluded from model fitting, feature selection, pruning-related fine-tuning, and fusion-model training, thereby preventing segment-level leakage between the training and test sets.

### 2.6 Ablation study

To evaluate the contribution of various components in our multimodal framework, we performed a series of ablation experiments. These were designed to evaluate the impact of; (i) each modality in isolation, (ii) pruning on model performance, (iii) different fusion strategies, and (iv) cross-modality interactions within specific fusion mechanisms.

Initially, we independently trained and evaluated each unimodal model utilizing the same LOSOCV procedure applied to the full multimodal system. This enabled us to measure the independent discriminative ability of each modality and discern the most discriminating information sources. These results also functioned as benchmarks for evaluating improvements in fusion. Subsequently, for each modality, we evaluated the performance of the original (unpruned) model against its pruned and fine-tuned counterpart. This analysis characterized the observed trade-off between model compression and classification performance across the evaluated sparsity levels. Subsequently, we methodically evaluated all five fusion procedures utilizing uniform validation configurations. The observed performance differences were used to examine how the evaluated fusion paradigms responded to inter-modality redundancy, complementarity, and noise within this cohort. To assess the resilience and adaptability of the fusion mechanisms, we conducted controlled ablations by sequentially eliminating one modality from the fusion process. This experiment was conducted for all five fusion procedures, elucidating which methods exhibit elegant degradation in the presence of absent or distorted modalities. Collectively, these ablation studies enhanced our comprehension of the impact of each architectural and algorithmic decision on overall performance, interpretability, and computational efficiency.

### 2.7 Statistical analysis

After screening multiple fusion strategies and modality combinations, four selected post hoc exploratory comparisons were conducted using paired participant-level out-of-fold predictions: LLMF vs. ABF using facial and upper-limb modalities; ABF vs. MTF using facial and gait modalities; ABF vs. IF using facial and gait modalities; and ABF face+gait vs. the clinical baseline. Differences in macro-averaged f1-score were evaluated by stratified paired bootstrap resampling with 5,000 iterations while preserving the observed class distribution. Percentile-based 95% confidence intervals were calculated for the paired differences. Exact McNemar tests were also performed using the paired correct/incorrect classifications. Holm adjustment was applied separately to the bootstrap and McNemar p values across the four comparisons. Although Holm adjustment was applied across these four comparisons, it did not account for the preceding screening of multiple fusion strategies and modality combinations. The resulting confidence intervals and adjusted p values should therefore be interpreted descriptively as post hoc exploratory analyses rather than as confirmatory tests of model superiority. Continuous clinical characteristics are presented as median [interquartile range]. Differences among the three fall-risk groups were assessed using the Kruskal–Wallis test. For disease duration, pairwise comparisons following the significant omnibus test were performed using two-sided Mann–Whitney U tests with Holm adjustment for multiple comparisons.

To enhance model interpretability, modality-specific attention weights were examined for the face + gait configurations of the ABF and MTF models. The “face + gait” configuration was selected because it was the highest-performing conventional learned fusion setting, was available for both ABF and MTF, and enabled a controlled comparison of modality reliance using the same clinically relevant inputs reflecting facial motor expression and locomotor function (see Section 3.4). Specifically, for every excluded participant, the modality weights produced by the relevant fusion model were preserved from the LOSOCV prediction. Attention weights were aggregated both collectively and individually for fall-risk classes A, B, and C, utilizing averages, standard deviations, medians, and bootstrap 95% confidence intervals derived from 5,000 resamples. Variations in face-attention weights among the three fall-risk classifications were assessed by permutation tests involving 10,000 iterations. Due to the fact that the face and gait weights total one, only the face weight was evaluated to prevent superfluous inference. The Holm correction was utilized in the ABF and MTF class-effect assessments.

Finally, to assess the incremental value of the multimodal digital biomarkers beyond conventional clinical information, we constructed a clinical baseline using age, sex, disease duration, levodopa-equivalent daily dose, and the MDS-UPDRS-III score in the ON-medication state after excluding item 3.12, which contributed to the definition of the clinical outcome. The clinical baseline was implemented using multinomial logistic regression and evaluated using participant-level leave-one-subject-out cross-validation. Within each fold, missing-value imputation and feature standardization were estimated exclusively from the training participants and subsequently applied to the held-out participant.

We additionally performed an exploratory analysis combining the clinical variables with the three ABF face+gait class-probability estimates. The ABF probabilities were precomputed out-of-fold estimates from the primary LOSOCV analysis. In each LOSO iteration of the combined model, multinomial logistic regression was fitted using the clinical variables and precomputed ABF probability vectors of the 146 training participants and was then applied to the corresponding variables of the held-out participant. However, the ABF probability vectors were generated in a separate LOSOCV procedure and were not recomputed within each outer training set of the combined model. Consequently, ABF models used to generate some training-participant probability vectors may have included the outer held-out participant. Complete exclusion of the held-out participant from every stage of the stacked pipeline therefore cannot be guaranteed, and the Clinical+ABF results were treated as exploratory rather than as an unbiased estimate of incremental performance.

## 3 Results

### 3.1 Clinical results and associations

A total of 147 patients with PD were included in the study. Their demographics and disease characteristics are presented in Table 2. The class distribution was moderately imbalanced, with 64 participants in class A, 44 in class B, and 39 in class C, corresponding to 43.5%, 29.9%, and 26.5% of the cohort, respectively. Thus, class A was more frequent than classes B and C, although all three groups retained sufficient representation for participant-level evaluation. Participants were grouped into three classes of cross-sectional fall-related vulnerability: (a) those without objectively detected postural instability during the ON-medication assessment (MDS-UPDRS item 3.12=0) and without any reported falls during the preceding six months; (b) those with postural instability (MDS-UPDRS item 3.12 ≥1) but no reported falls during the preceding six months; and (c) those reporting at least one fall during the preceding six months, irrespective of their MDS-UPDRS item 3.12 score.

**Table 2:** Clinical characteristics across the three fall-risk groups.

Variable	Overall	A	B	C	*p*-value
Sample size (n)	147	64	44	39	–
Age	66.48 [59.18–72.06]	64.82 [57.47–70.23]	68.55 [60.58–72.32]	67.76 [63.63–73.41]	0.039
Age at onset	58.70 [51.24–65.07]	58.55 [50.92–63.61]	59.97 [51.88–66.56]	56.00 [51.00–64.21]	0.647
Disease duration (years)	6.00 [3.00–10.04]	5.00 [2.67–7.85]	6.12 [2.58–9.70]	8.73 [4.33–14.85]	0.0095
LEDD (mg/day)	618.00 [400.00–914.00]	500.00 [307.50–751.75]	736.00 [403.50–957.75]	740.00 [495.00–1072.50]	0.005
MMSE	28.00 [26.00–29.00]	28.00 [26.00–29.00]	28.00 [26.00–29.00]	27.00 [25.00–28.00]	0.167
UPDRS total	51.00 [37.00–75.00]	41.00 [30.00–61.00]	50.00 [37.00–75.25]	70.00 [51.50–84.00]	0.00002
UPDRS-III (ON)	28.00 [20.50–39.00]	24.00 [17.00–31.50]	30.00 [24.00–39.50]	33.00 [26.00–43.00]	0.00089
UPDRS-III (OFF)	39.00 [28.00–50.00]	33.00 [25.00–43.00]	39.00 [31.75–52.50]	43.00 [32.00–52.00]	0.0068
UPDRS-IV	2.00 [0.00–7.00]	1.00 [0.00–4.25]	2.00 [0.00–8.00]	4.00 [1.00–7.50]	0.0298
NMSS total	56.00 [38.50–96.25]	44.00 [24.50–74.50]	57.00 [39.50–105.50]	84.50 [48.75–133.50]	0.00045
PDQ-39 total	18.54 [8.59–32.19]	11.51 [6.55–20.23]	18.85 [9.17–36.51]	31.43 [20.82–40.17]	0.0000045

Values are reported as median [IQR].

Age differed across the three groups in the omnibus analysis (p=0.039). The median age was 64.82 years in class A, 68.55 years in class B, and 67.76 years in class C. Because post hoc pairwise tests were not performed for age, no pairwise group differences are inferred. Disease duration also differed significantly among the three groups (Kruskal–Wallis H(2)=9.304, p=0.0095), with the highest median disease duration observed in class C. Holm-adjusted pairwise comparisons showed that disease duration was significantly longer in class C than in class A (p=0.008), whereas the comparisons between classes A and B (p=0.269) and between classes B and C (p=0.132) were not statistically significant. The median values of several motor-severity measures increased descriptively from class A to classes B and C, and their omnibus tests indicated overall differences among the three groups. This pattern was evident in MDS-UPDRS-III scores (in both the ON- and OFF-medication states) and in MDS-UPDRS-IV, with the latter reflecting the extent of motor complications, including motor fluctuations and dyskinesias.

NMSS and PDQ-39 scores also differed in the omnibus analyses, with descriptively higher median values in classes B and C than in class A. This trend indicated a broader escalation in PD-related parameters, while LEDD differed across the three groups in the omnibus analysis (p=0.005), with median values of 500.00, 736.00, and 740.00 mg/day in classes A, B, and C, respectively. Because post hoc pairwise tests were not performed for LEDD, these descriptive differences should not be interpreted as significant pairwise group differences.

Together, these findings reinforce the clinical relevance of our grouping strategy. Although balance impairment and fall history were the sole classification criteria, the resulting groups showed descriptive differences in several motor, non-motor, and quality-of-life measures, mirroring what is commonly observed in clinical practice as PD advances.

### 3.2 Model pruning

Figure 2 illustrates the variation in f1-score for each modality at pruning levels ranging from 10 to 90%. The gait model exhibited notable resilience to pruning, sustaining f1-scores exceeding 79% at 70% sparsity and reaching 83.06% at 50% pruning. Because pruning levels were selected retrospectively from aggregate LOSOCV results rather than within a fully nested model-selection procedure, these performance estimates should be considered exploratory and may be affected by selection optimism. The face and upper limb models exhibited a progressive deterioration in performance as pruning intensified, with more significant declines occurring beyond 60% and 50% sparsity, respectively. The speech model demonstrated heightened sensitivity, with performance varying between 45% and 54% across pruning levels, showing no discernible advantage from moderate pruning. In general, gait demonstrated the most resilience to severe pruning, but speech model performance was the most adversely impacted. These findings indicate modality-specific differences in tolerance to structured pruning. However, because the pruning levels were compared using aggregate LOSOCV results rather than nested model selection, the selected configurations and their associated performance estimates may be optimistic. Future studies should determine pruning levels within an independent or fully nested model-selection procedure.

**Figure 2:**
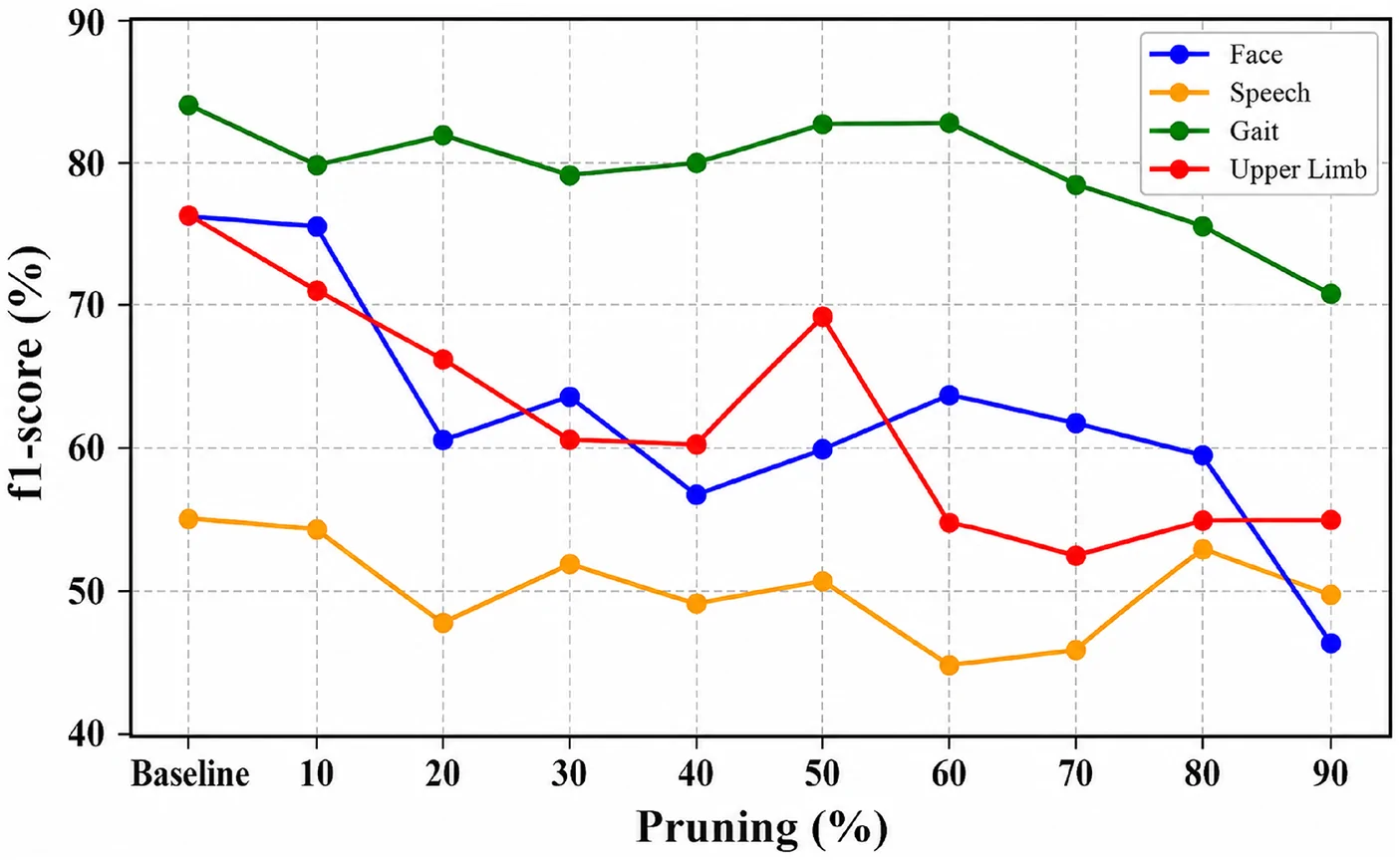
f1-score percentage changes with different levels of pruning. Performance generally declines with higher pruning, though the degree of decline varies across modalities.

Figure 3 presents the effect of pruning on inference latency (ms/sample) and estimated computational cost (Giga floating-point operations per second—GFLOPs) across modalities. Inference time consistently diminished with the escalation of pruning across all models. The face model demonstrated the most significant absolute reduction, decreasing from 24.91 ms/sample at baseline to 17.62 ms/sample after 90% pruning, whilst the gait model diminished from 15.47 to 10.31 ms/sample. The upper limb and speech models demonstrated negligible absolute change owing to their already low baseline latencies (1.24 ms and 0.138 ms, respectively). GFLOPs exhibited a comparable pattern, with incremental decreases aligned with the pruning ratio. The gait model’s GFLOPs reduced from 2.66 to 1.49, while the face model declined from 0.19 to 0.13 between the baseline and 90% pruning. Modifications in the upper limb and speech models were comparatively minimal, indicating their lightweight structures.

**Figure 3:**
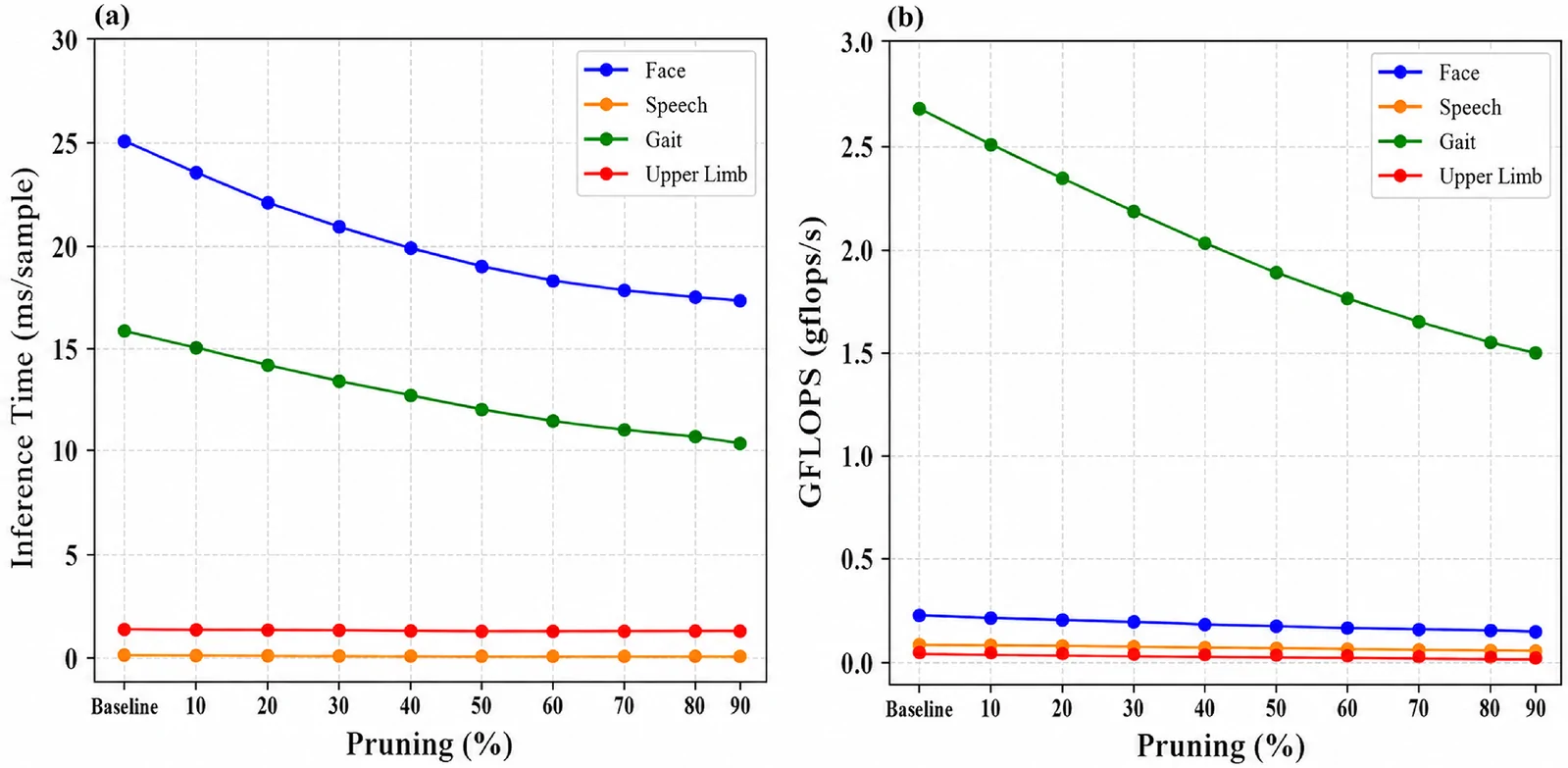
Comparison of inference time and estimated computational cost across pruning levels. Structured pruning generally reduced inference latency and GFLOPs as pruning increased. The measurements were obtained exclusively in the study’s high-performance computing environment. They do not demonstrate performance, feasibility, or deployment readiness on wearable, mobile, edge, or other resource-constrained hardware.

Figure 4 illustrates the percentage reduction in model size for each modality across pruning levels from 10 to 90%. All four models exhibited uniform compression tendencies, with the speech and face models displaying the most significant decreases. With 90% pruning, the speech model attained a 96.80% reduction in size, whilst the face model achieved a similar reduction of 92.02%. The gait model showed a more mild compression, with a 33.90% decrease at 90%, whilst the upper limb model displayed diminished sensitivity to pruning, obtaining merely a 6.34% reduction at the maximum sparsity level. The disparities indicate architectural differences: for instance, the speech and upper limb models are considerably lighter at baseline (325 KB and 275 KB, respectively) than the face (408.4 MB) and gait (2.3 MB) models. Significantly, even little pruning (e.g., 30%) resulted in substantial savings in the face model (35.15%) and particularly in the voice model (50.56%), with negligible effects on classification performance (see Figure 2). This illustrates the efficacy of structured pruning in diminishing memory usage while maintaining accuracy, especially in highly redundant designs.

**Figure 4:**
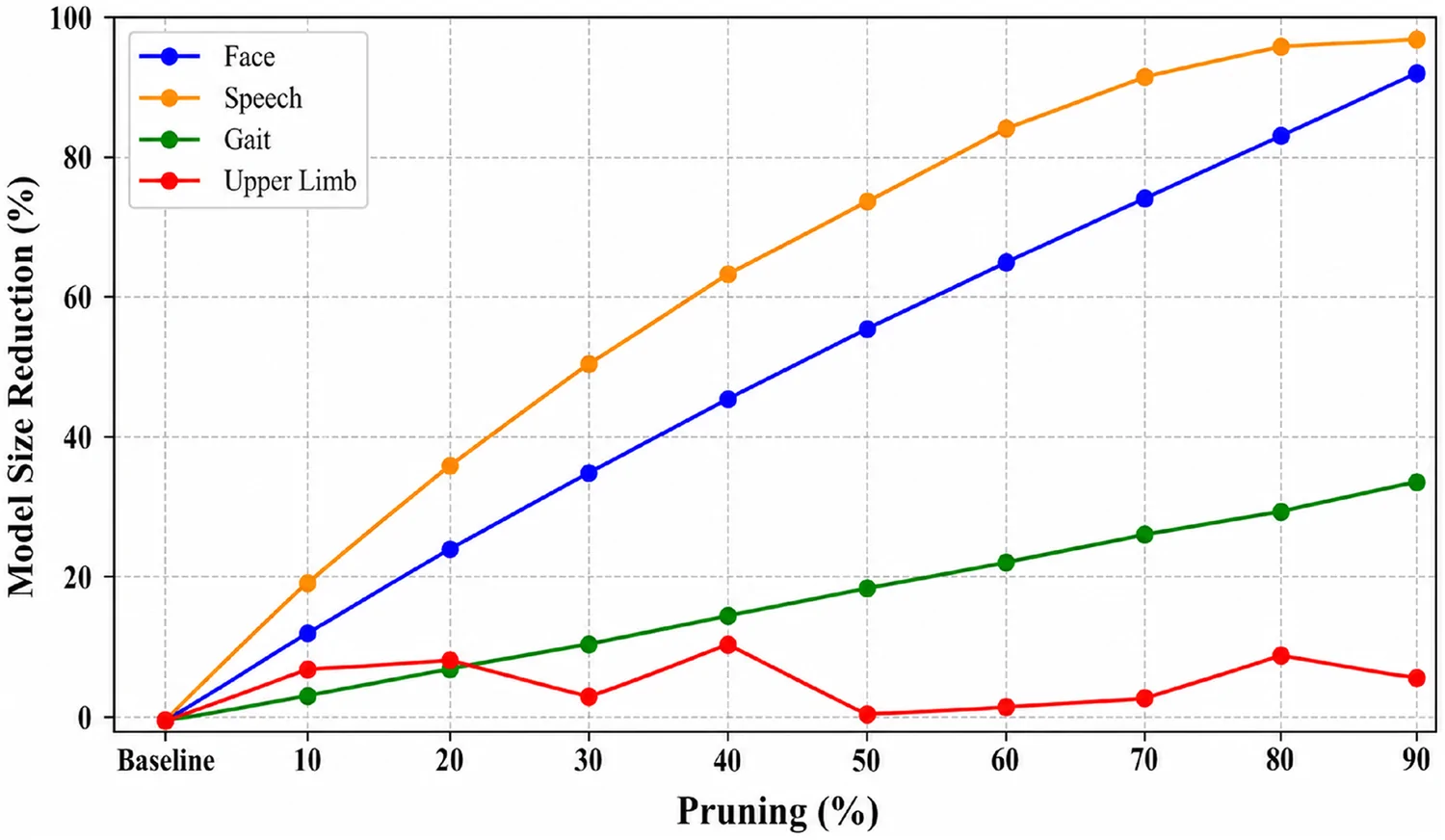
Percentage decrease in model size for each modality as pruning ratios increase from 10 to 90%.

### 3.3 Selection of pruned model configurations

To determine the pruned configurations for the fusion experiments, we evaluated the trade-off among classification performance (f1-score), computational efficiency (inference time and GFLOPs), and memory conservation (model size reduction). In the face modality, pruning at 10% resulted in a significant size reduction 12.21% and a latency decrease 5.62%, while performance remained at baseline levels (75.29% vs. 76.32% f1). The gait model exhibited substantial resilience to pruning, with 50% sparsity resulting in only a marginal decrease in the f1-score (83.06% compared with the baseline value of 83.45%), together with an 18.71% reduction in model size and a 23.77% reduction in latency; hence, the 50% pruned configuration was selected. In speech, pruning at 80% achieved a substantial size reduction 95.61% with minor alteration in the f1-score relative to the baseline (54.16% against 54.87%), and slight increase in inference time. In the upper limb modality, 50% pruning enhanced latency by 12.92% with a slight decrease in f1-score (69.43% compared to 76.59%) and negligible GFLOPs, and it was therefore selected as the working configuration for subsequent fusion analyses. Despite conducting additional tests with varying pruning levels for each modality, consistent enhancements in performance and efficiency measures were not achieved. Consequently, the chosen configurations, Face (10%), Gait (50%), Speech (80%), and Upper Limb (50%), were utilized in all evaluations of the fusion strategies (Figure 5 and Figure S1 in Suppl.). Accordingly, the selected pruning configurations and the fusion analyses based on them should be interpreted as exploratory with respect to post-selection performance.

**Figure 5:**
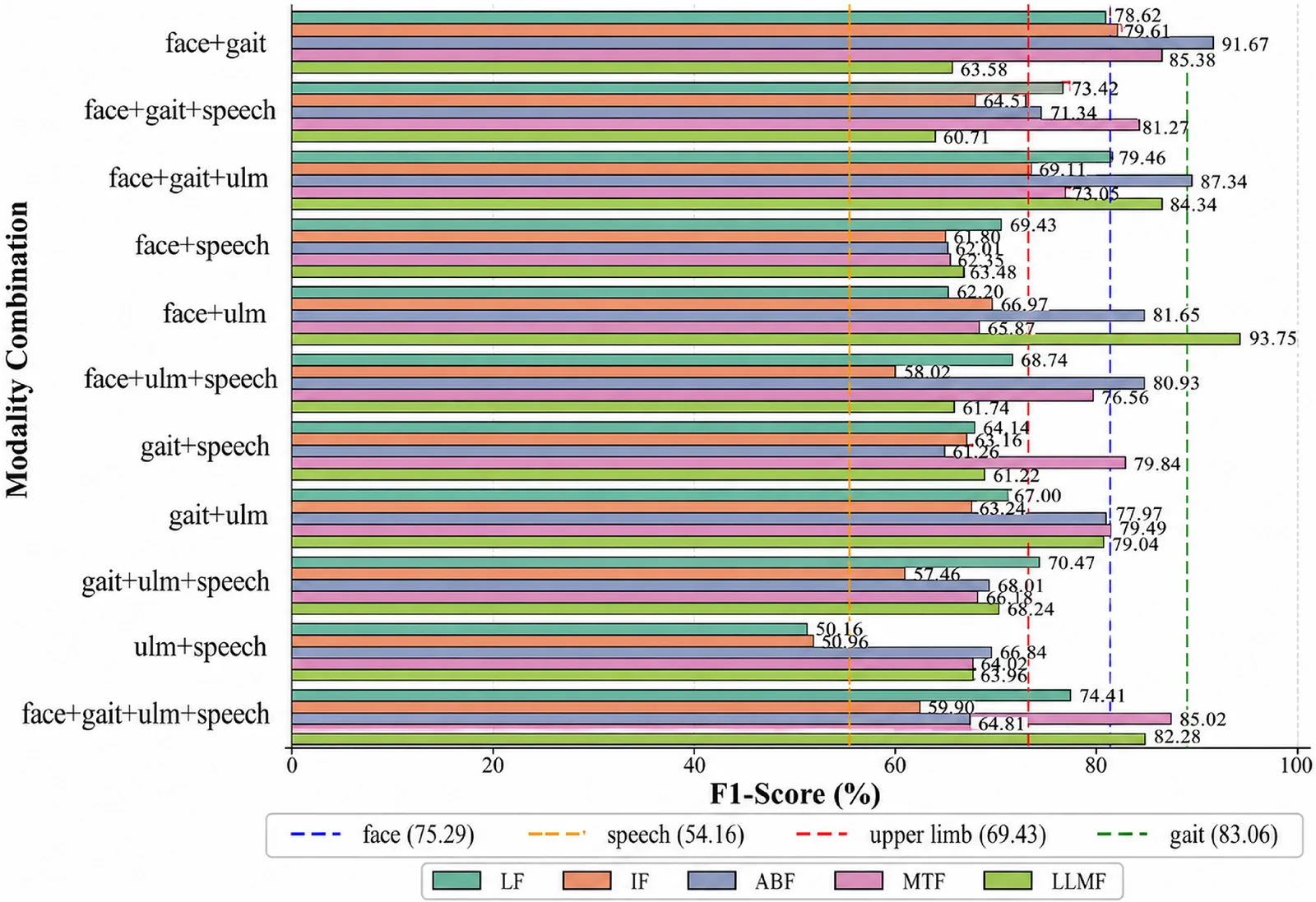
Observed macro-F1 scores for the evaluated multimodal fusion strategies across combinations of facial, gait, speech, and upper-limb modalities. Grouped bars represent the fusion strategies, while dashed vertical lines indicate the performance of the selected pruned unimodal models. The LLMF values are exploratory single-run results based on one recorded API call per participant–modality configuration. The API used a mutable model alias, and repeated-run stability and exact model-version reproducibility were not established.

### 3.4 Fusion and modality ablation study

The LF exhibited enhancements in performance across several modality combinations (Figure S2 in Suppl.). The combination of face and gait (“face + gait”) achieved f1-score 78.62%, above that of the standalone face model (76.19% f1), while the incorporation of the upper limb modality further enhanced the score to 79.46% f1. Nonetheless, combinations that included speech frequently diminished overall performance, exemplified by “face + gait + speech” at 73.42% f1. The comprehensive four-modality LF fusion achieved 74.41% f1, which did not surpass the independent gait model.

IF yielded lower scores than the selected pruned unimodal models in most configurations (Figure S3 in Suppl.). Among the IF configurations evaluated, “face + gait” produced the highest observed macro-f1 of 79.64%, slightly exceeding the facial unimodal model. However, the remaining configurations, including the four-modality fusion at 59.90%, did not exceed the corresponding unimodal baselines. Because multiple modality combinations were evaluated, the highest observed value should be interpreted descriptively rather than as an unbiased estimate of the optimal IF configuration.

ABF produced high observed performance in selected modality configurations (Figure S4 in Suppl.). The “face + gait” and “face + gait + ulm” configurations achieved macro-f1 values of 91.67% and 87.34%, respectively, exceeding the corresponding unimodal results in the present analysis. These values were obtained after evaluation of multiple modality combinations and should therefore not be interpreted as definitive evidence that these configurations are universally superior.

The clinical baseline, which included age, sex, disease duration, levodopa-equivalent daily dose, and MDS-UPDRS-III in the ON-medication state after exclusion of item 3.12, achieved a macro-f1 of 50.10%, a macro-averaged one-vs.-rest ROC-AUC of 70.20%, and a macro-averaged PR-AUC of 53.30% (Table 3). The ABF face+gait model achieved substantially higher corresponding values of 91.67%, 89.74%, and 86.55%, respectively. A post hoc exploratory paired comparison between the ABF face+gait model and the clinical baseline is reported in Table 4.

**Table 3:** Performance of the clinical baseline, the ABF face+gait digital model, and the exploratory Clinical + ABF model.

Model	Macro-f1 (%)	Macro ROC-AUC (%)	Macro PR-AUC (%)	Brier score	ECE
Clinical baseline	50.10 [42.0–58.1]	70.20 [63.2–76.8]	53.30 [45.1–61.2]	0.37 [0.31–0.43]	0.09 [0.05–0.14]
ABF face + gait	91.67 [86.8–95.5]	89.74 [84.7–94.1]	86.55 [80.6–91.7]	0.21 [0.16–0.27]	0.05 [0.03–0.09]
Clinical + ABF	94.70 [90.5–97.5]	93.10 [89.1–96.4]	88.70 [83.3–93.3]	0.20 [0.15–0.25]	0.04 [0.02–0.07]

Values in brackets represent bootstrap 95% confidence intervals based on 5,000 participant-level resamples. Higher values indicate better performance for macro-f1, ROC-AUC, and PR-AUC, whereas lower values indicate better calibration for the Brier score and expected calibration error (ECE). The Clinical+ABF results should be interpreted as exploratory because the stacking pipeline was not evaluated using fully nested cross-fitting.

**Table 4:** Post hoc exploratory paired comparisons between selected models and fusion strategies.

Comparison	Δ macro-f1	95% CI	Bootstrap p	McNemar p
LLMF face + ulm vs. ABF face+ulm	+12.1	[4.2, 19.3]	0.008	0.028
ABF face+gait vs. MTF face+gait	+6.3	[−0.4, 12.5]	0.095	0.157
ABF face+gait vs. IF face+gait	+12.1	[5.1, 18.4]	0.004	0.019
ABF face+gait vs. clinical baseline	+41.6	[32.0, 50.5]	<0.001	<0.001

Values represent post hoc exploratory paired comparisons based on participant-level out-of-fold predictions. The models and modality combinations were selected after screening multiple fusion strategies and configurations. Bootstrap and McNemar p values were Holm-adjusted across the four comparisons shown; however, this adjustment did not account for the preceding model and configuration screening. The results should therefore be interpreted descriptively and not as confirmatory evidence of model superiority. A Clinical + ABF vs. ABF comparison was not included because the stacking pipeline was not evaluated using fully nested cross-fitting. The LLMF comparison additionally reflects a single recorded API run per participant–modality configuration; repeated-run stability and exact model-version reproducibility were not established.

**Table 5:** Modality-level attention weights for “face + gait” fusion.

Fusion	Class	Modality	Mean	SD	95% CI
ABF	Overall	Face	0.555	0.308	[0.500, 0.610]
ABF	Overall	Gait	0.445	0.308	[0.389, 0.500]
ABF	A	Face	0.553	0.289	[0.474, 0.631]
ABF	B	Face	0.546	0.330	[0.437, 0.652]
ABF	C	Face	0.569	0.325	[0.454, 0.680]
MTF	Overall	Face	0.981	0.037	[0.974, 0.987]
MTF	Overall	Gait	0.019	0.037	[0.013, 0.026]
MTF	A	Face	0.983	0.040	[0.970, 0.992]
MTF	B	Face	0.978	0.038	[0.965, 0.990]
MTF	C	Face	0.981	0.033	[0.968, 0.991]

In an exploratory analysis, combining the clinical variables with the ABF out-of-fold class-probability estimates yielded a macro-f1 of 94.70%, a macro ROC-AUC of 93.10%, and a macro PR-AUC of 88.70%. The combined model also showed numerically lower calibration-error estimates than ABF alone, with a Brier score of 0.20 vs. 0.21 and an ECE of 0.04 vs. 0.05. However, because the Clinical+ABF stacking pipeline was not evaluated using fully nested cross-fitting, these differences may be optimistic and should not be interpreted as confirmatory evidence that Clinical+ABF outperforms ABF alone.

MTF produced relatively similar observed performance across several modality combinations (Figure S5 in Suppl.). The combination of “face + gait” yielded 83.38%, nearly equivalent to the standalone gait model, whereas “face + gait + speech” attained f1-score 81.27%, and the comprehensive four-modality fusion achieved 85.02% f1. “gait + speech” (79.84% f1) and “gait + ulm” (79.99% f1) exhibited consistent performance, with f1-scores remaining close across different modality pairings, indicating limited sensitivity to the specific modality combination.

In the exploratory single-run LLMF analysis, 1,617 API calls were attempted and succeeded. The face+upper-limb configuration yielded a macro-F1 of 93.75% based on n=147 participants, whereas gait + upper limb and the four-modality configuration yielded macro-F1 values of 79.04% (n=147) and 82.28% (n=147), respectively. Face + gait and face + gait + speech yielded macro-F1 values of 71.34% (n=147) and 60.71% (n=147), respectively. Each value represents one recorded API run per participant–modality configuration; repeated-run stability and exact model-version reproducibility were not established.

Overall, the ablation across several fusion strategies revealed that the impact of modality integration significantly differed based on the strategy employed and the particular combination of modalities utilized. As seen in Figure 5, various fusion configurations, especially ABF and LLM, exceeded the performance of independent baselines, although others diminished efficacy relative to the single-modality pruned models. ABF produced higher observed macro-F1 values than the corresponding unimodal baselines in selected configurations, particularly face+gait and face + gait + upper limb. These configurations were identified after evaluating multiple fusion strategies and modality combinations and should not be interpreted as independently confirmed or reproducibly superior. The exploratory LLMF face+upper-limb configuration produced the highest numerical macro-f1 in the recorded single-run analysis. However, because the LLM analysis lacked an immutable model snapshot, fixed inference parameters, and repeated-run assessment, it cannot be regarded as a reproducibly superior, clinically validated, or best-performing configuration beyond the recorded exploratory run. MTF attained the highest f1-score (85.02%) by integrating all four modalities.

The post hoc exploratory paired comparison of the recorded single-run outputs showed a 12.1 percentage-point macro-F1 difference between LLMF and ABF using facial and upper-limb modalities (95% bootstrap CI: 4.2–19.3; Holm-adjusted bootstrap p=0.008; Holm-adjusted McNemar p=0.028). This comparison characterizes the recorded outputs only and should not be interpreted as evidence of reproducible superiority. In another post hoc exploratory comparison, the observed macro-F1 for ABF exceeded that for IF using facial and gait modalities by 12.1 percentage points (95% CI: 5.1–18.4; Holm-adjusted bootstrap p=0.004; Holm-adjusted McNemar p=0.019). The observed 6.3 percentage-point difference between ABF and MTF using facial and gait modalities had a confidence interval that included zero (95% CI: −0.4–12.5; Holm-adjusted bootstrap p=0.095; Holm-adjusted McNemar p=0.157). Because these models and configurations were selected after screening multiple alternatives, all comparisons are exploratory and do not provide confirmatory evidence of model superiority.

In the selected post hoc exploratory comparison, ABF face+gait had a higher observed macro-F1 than the clinical baseline. The paired macro-f1 difference was 41.6 percentage points (95% bootstrap CI: 32.0–50.5; Holm-adjusted bootstrap p<0.001; Holm-adjusted McNemar p<0.001). This observed difference applies to the evaluated out-of-fold predictions within this cohort and should not be interpreted as confirmatory evidence that the multimodal model is generally superior to clinical assessment.

The attention analysis revealed distinct modality-reliance patterns between the two fusion architectures. For ABF using facial and gait modalities, the mean attention weight was 0.555 for face (95% CI: 0.500–0.610) and 0.445 for gait (95% CI: 0.389–0.500), indicating relatively balanced integration of the two modalities. The substantial participant-level variability (SD = 0.308 for both modalities) indicates that ABF varied its relative reliance on facial and gait information across participants. Mean facial attention weights were similar across classes A, B, and C (0.553, 0.546, and 0.569, respectively), with no evidence of a class-related difference ($\eta^{2}=0.00085$; Holm-adjusted permutation p=1.000). Attention weights were interpreted as indicators of model reliance rather than as measures of causal or clinical importance.

By contrast, MTF allocated nearly all attention to the face modality, with an overall mean face weight of 0.981 (95% CI: 0.974–0.987) and a mean gait weight of 0.019 (95% CI: 0.013–0.026). This strong concentration was consistent across classes A, B, and C, and the class-related effect was not significant ($\eta^{2}=0.00219$, Holm-adjusted permutation p=1.000). These findings indicate that ABF performed adaptive multimodal integration, whereas MTF exhibited a pronounced preference for facial information. Attention weights are interpreted as indicators of model reliance rather than causal clinical importance.

### 3.5 Hardware and software configuration

All experiments were conducted on a high-performance GPU cluster running Ubuntu 22.04.5 LTS (Jammy Jellyfish) with Python 3.9.21, PyTorch 2.5.1, and CUDA 12.4. The system was equipped with dual AMD EPYC 7742 64-core processors (256 hardware threads in total) and 1.0 TiB of system RAM. GPU acceleration was provided by eight NVIDIA A100-SXM4 units, each with 40 GB of high-bandwidth memory, connected via NVLink. The environment leveraged CUDA 12.4 for GPU computation. In multimodal experiments, unimodal models were executed in parallel on separate GPUs rather than sequentially, allowing efficient fusion without increasing end-to-end latency.

## 4 Discussion

This study developed a multimodal framework for cross-sectional stratification of fall-related vulnerability in patients with PD. The clinical endpoint was defined using current postural instability and fall history during the preceding six months. Conventional metrics derived from gait and limb movement capture axial and appendicular motor impairment, but fail to account for dysfunction in non-motor circuits. The innovation of our study lies in integrating traditional motor data streams, such as gait and upper limb movement, with non-traditional signals derived from facial expression and speech into a unified model. In the present cohort, selected fusion configurations that incorporated facial information yielded higher observed classification performance than the corresponding unimodal or alternative multimodal configurations, whereas the contribution of speech was smaller and less consistent. These findings suggest that facial and, to a lesser extent, speech signals may contain complementary cross-sectional information not fully represented by gait and upper-limb measurements.

One possible explanation is that facial expressivity and speech are associated with several motor and non-motor manifestations of PD, including hypomimia, dysarthria, hypophonia, axial dysfunction, cognitive status, mood, and medication effects. However, the present analysis did not test the underlying biological mechanisms responsible for the observed model performance. It therefore cannot determine whether facial or speech features directly reflect mechanisms involved in falls, nor whether their contribution is independent of overall disease severity or other correlated clinical characteristics. Accordingly, the observed added value should be interpreted as model-dependent complementary discriminative information within this cohort. It does not independently validate facial or speech measures as clinical biomarkers and does not establish a causal relationship between these signals and balance impairment or falls.

From a technical perspective, the study evaluated modality-specific pruning and multimodal fusion techniques for cross-sectional classification of fall-related vulnerability among independently ambulatory participants with PD assessed in the ON-medication state. Through structured pruning of models across four input domains, facial video, gait, speech, and upper-limb kinematics, we identified reduced configurations with favorable observed trade-offs among classification performance, inference efficiency, and model compactness within the experimental environment. The selected compressed models served as the foundation for fusion experiments, in which several integration procedures were evaluated against unimodal baselines. Across multiple fusion strategies, selected multimodal configurations yielded higher observed classification performance than the corresponding pruned unimodal models while retaining reduced inference latency and model size. These findings demonstrate computational efficiency within the experimental environment but do not establish deployment readiness, prospective clinical utility, or performance on resource-constrained devices.

Pruning results indicated that significant model compression may be attained without severely degrading classification performance. The speech model could be pruned by 80% while maintaining an f1-score close to baseline, leading to a 95% reduction in size and minimal computational overhead. Likewise, the selected gait configuration retained similar observed classification performance at 50% pruning while reducing model size and computational requirements. The pruning results indicate that substantial model compression can be achieved while retaining similar classification performance in selected configurations. In the present study, however, pruning was not evaluated using external data, and no conclusion regarding improved generalization can therefore be drawn. Previous studies have reported that pruning may affect generalization through reduced model complexity (40–42).

The fusion results demonstrated significant variability in the interaction of modalities across various fusion methodologies. Late fusion yielded marginal enhancements when integrating highly predictive modalities, such as face and gait (78.62% f1-score compared to unimodal baselines); however, performance deteriorated upon the indiscriminate inclusion of speech or upper limb signals, reflecting established constraints of naïve LF, which overlooks signal-level interactions and assumes uniform reliability across modalities (43). IF produced variable outcomes, frequently falling short of the independent models’ performance, likely due to imbalances in feature dimensionality across modalities, where simple concatenation causes higher-dimensional representations to dominate the fused space, alongside scale discrepancies—a recognized limitation of naïve feature-level fusion in multimodal learning (43). ABF produced higher observed macro-F1 values than the corresponding unimodal baselines for selected combinations, including face+gait (91.67%) and face + gait + upper limb (87.34%). These results indicate that the evaluated attention mechanism combined complementary signals effectively within this cohort, although external reproducibility and clinical utility were not assessed (44). The paired comparison with the clinical baseline indicated that the face+gait ABF model provided greater cross-sectional discrimination than a multinomial logistic-regression model incorporating age, sex, disease duration, medication dosage, and motor severity after exclusion of the postural-stability item. However, the exploratory clinical + ABF analysis was not implemented using fully nested cross-fitting. Consequently, the small numerical improvement observed after adding clinical variables to ABF cannot be interpreted as an unbiased estimate of incremental benefit.

The strong performance of the “face + gait” combination is clinically and computationally plausible because the two modalities capture partly distinct manifestations of Parkinsonian dysfunction. Gait directly reflects locomotor control, balance, postural stability, stride regulation, and movement variability, all of which are closely related to fall vulnerability. Facial expression, by contrast, reflects cranial and axial motor dysfunction, including hypomimia and reduced motor expressivity, and may also encode aspects of cognitive and emotional regulation that are not fully represented by gait. Therefore, the two modalities provide complementary rather than redundant information. This interpretation is supported by the attention analysis of the “face + gait” ABF model, which assigned mean weights of 0.555 to face and 0.445 to gait, with substantial participant-level variability. Meanwhile, multimodal transformer fusion exhibited strong performance, particularly in the integration of gait and upper limb data, by modeling global dependencies across modality-specific embeddings through a transformer encoder that aligns and consolidates complementary temporal-spatial signals; this transformer-based fusion has also enhanced performance in clinical prediction tasks involving structured clinical and textual data (45). Among the MTF configurations evaluated, the four-modality combination yielded the highest observed macro-f1 of 85.02%. This finding suggests that MTF may accommodate information from larger modality sets, although the observed maximum should be interpreted cautiously because several modality combinations were compared.

LLMF represents a distinct exploratory decision-level fusion approach in which modality-specific predictions were organized into a structured prompt containing class probabilities, segment counts, and confidence information (46). In the recorded single-run analysis, performance varied substantially across modality combinations, with the face+upper-limb configuration yielding the highest numerical macro-f1 of 93.75%. These findings should be interpreted cautiously. The LLM analysis used a mutable model alias, generation parameters were not explicitly fixed, and each participant–modality configuration was queried only once. Therefore, repeated-run stability and exact model-version reproducibility were not established. Furthermore, the generated rationales and optional modality weights were not validated as faithful explanations of the classification decisions. Accordingly, the LLMF results are presented solely as an exploratory proof-of-concept and do not demonstrate expert-level reasoning, state-of-the-art performance, clinical validation, or reproducible superiority over conventional fusion methods.

In contrast to typical multimodal approaches in PD research (Table 6), this study varies in its clinical aims and methodological breadth. Earlier research has primarily concentrated on the binary differentiation between individuals with Parkinson’s disease and healthy subjects or on overarching disease-severity categorization utilizing a blend of gait, speech, upper-limb motion, tapping, and cognitive assessments (47–50). Butt et al. (47) documented a f1-score of 84.20% by amalgamating gait and upper-limb data via feature-level fusion, whereas Lim et al. (50) achieved a f1-score of 80.56% utilizing gait, speech, and tapping signals through LF.

**Table 6:** Comparison of representative multimodal approaches for PD and the present study, highlighting differences in clinical target, modality combination, fusion strategy, and methodological contribution.

Ref.	Subjects	Clinical target	Model	Performance	Modality combination	Fusion	Main distinction
Butt et al. (47)	64 PD/50 HC	PD vs. HC	BiLSTM	f1 = 84.20%	gait + ulm	Feature-level	Binary diagnosis using two movement modalities
Vásquez-Correa et al. (48)	44 PD / 40 HC	PD severity	CNN + SVM	Accuracy = 97.60%	gait + speech + ulm	LF	Severity assessment using static decision-level fusion
Prince et al. (49)	87 PD/46 HC	PD vs. HC	CNN + Ensemble	Accuracy = 82.00%	gait + speech + memory + tapping	LF	Binary diagnosis using motor, speech, and cognitive tasks
Lim et al. (50)	213 PD/283 HC	PD vs. HC	SVM	f1 = 80.56%	gait + speech + tapping	LF	Smartphone-derived multimodal binary classification
This work	147 PD	Three-class	See Section 2.3	f1 = 91.67% f1 = 87.34% f1 = 85.02% f1 = 93.75%	face + gait face + gait + ulm face + gait + speech + ulm face + ulm	ABF ABF MTF LLMF	Cross-sectional classification in a PD-only cohort using four modalities, five fusion strategies, structured pruning, and modality ablation

${}^{a}$Exploratory LLMF result based on one recorded API run per participant–modality configuration. An immutable model snapshot was not recorded, generation parameters were not explicitly fixed, and repeated-run stability was not assessed. The value is therefore descriptive of that recorded run only and should not be interpreted as a stable performance estimate, a reproducible benchmark, evidence of superiority over ABF or other fusion methods, or evidence of clinical effectiveness or validation.

Conversely, the current research focuses on three-tiered fall-risk categorization within a group solely consisting of patients with Parkinson’s Disease, instead of differentiating between diagnosed individuals and healthy subjects. Selected configurations produced observed macro-F1 values of 91.67% for ABF with facial and gait data and 85.02% for MTF with all four modalities. The face + upper-limb LLMF configuration produced a numerical macro-F1 of 93.75% in the exploratory single-run analysis; this value is reported descriptively and cannot be interpreted as a reproducible benchmark or as evidence of superiority. The performance of the conventional fusion configurations may reflect two principal considerations. Initially, face expressiveness offers supplementary insights into axial motor impairment and possibly non-motor symptoms that might not be entirely captured by gait or upper-limb assessments alone. Secondly, ABF and LLMF possess the capability to adjust the input from each modality based on its assessed significance or reliability, in contrast to fixed averaging or straightforward feature amalgamation. Nonetheless, direct numerical comparisons must be approached with caution due to variations in clinical outcomes, cohort characteristics, class definitions, input tasks, and validation methodologies among the research. Consequently, the findings do not establish superiority over earlier systems. They show that selected multimodal configurations provided useful cross-sectional discrimination among the prespecified fall-related vulnerability groups within this cohort.

Clinically, the relative performance of modalities aligns with existing knowledge on fall risk in PD. Gait abnormalities, including diminished stride length and velocity, and postural instability are among the most significant predictors of falls (51, 52). Arm swing, likewise, contributes meaningfully to dynamic balance and postural control (53); although PD patients can partially compensate for reduced and/or asymmetric arm swing through adjustments in lower-limb and trunk mechanics, these adaptive strategies may not fully counterbalance the potential increase in fall vulnerability. In our work, gait and upper limb signals exhibited the most reliable unimodal performance and contributed to fusion configurations with comparatively high observed performance in this cohort.

Although the relationship between facial expression and falls of any etiology has never been explored in PD, prior work has shown that hypomimia correlates with more severe axial motor signs after adjusting for disease duration, sex, and dopaminergic therapy (54). Interestingly, incorporation of the facial modality, despite its indirect connection to fall mechanics, provided additional value. In contrast, speech contributed less consistently to classification performance. While both hypomimia and speech impairment may reflect a mixture of motor and non- motor dysfunction, particularly cognitive impairment, the etiologic basis of the latter is broader and often non-PD- specific. Hypomimia tends to respond well to dopaminergic therapy (23, 54), at least partly, reinforcing its role as a core parkinsonian feature and its alignment with other PD-related dopaminergic symptoms. Speech impairment, on the other hand, tends to be less responsive to dopaminergic medication and may progress independently of global motor decline, supporting the assumption of a non-dopaminergic substrate (55, 56). Additionally, approximately 15% of older community-dwelling individuals present with voice abnormalities attributed to age-related structural changes of the larynx and/or vocal folds (presbyphonia), pulmonary dysfunction, comorbidities, menopause, medications, dentures and others (57, 58). Moreover, “talking while walking” has been highlighted as a challenging process for PD patients due to difficulties in multi-tasking. Although it has been suggested that high cognitive or linguistic load—such as the effort required by a PD patient with hypophonia or other speech issues to be understood—while walking may increase fall risk, particularly under demanding situations (e.g., stairs or poor lighting) (59), this association has not been confirmed (60).

Our results underline that gait and upper-limb movement parameters provided the most robust cross-sectional discrimination among the fall-related vulnerability groups, likely reflecting their more direct and well-characterized motor underpinnings. Although expanding estimation models to incorporate additional facets of PD phenomenology is challenging, it also holds great potential. Each modality captures a distinct dimension of the disorder, contributing unevenly to disease burden. Multimodal systems can combine these complementary aspects of motor and non-motor phenomenology, improving diagnostic accuracy across different patient subgroups and enabling a more individualized understanding of fall vulnerability. This approach is particularly valuable given the heterogeneity of PD, where different symptoms may dominate the clinical picture at different stages or in different individuals. In our cohort, although participants were classified solely on the basis of balance impairment and fall history, the resulting groups naturally reflected a continuum of PD progression, aligning with established clinical parameters such as disease duration, larger motor and non-motor burden, and higher dopaminergic treatment requirements. While integrating diverse modalities requires careful clinical contextualization, the present findings suggest that multimodal information may provide a more comprehensive cross-sectional characterization of fall-related vulnerability than individual modalities alone. Ultimately, multimodal AI-based evaluation may provide a research framework for cross-sectional risk stratification and may inform the design of future clinical assessment studies.

These findings have potential implications for future clinical research and technology development. Multimodal acquisition could potentially support future remote and unobtrusive assessment: facial and vocal cues may be recorded using standard smartphone cameras and microphones, while gait and upper-limb movements may be captured using commonly accessible consumer wearables. However, the present study did not evaluate the complete multimodal pipeline in real-world or home-based settings. These technologies may therefore support future investigation in such settings (61, 62). The observed reductions in model size and computational cost motivate future testing on representative edge devices to determine whether similar classification performance can be maintained under resource constraints (63). Future edge implementation would depend not only on model dimensions but also on end-to-end latency, memory utilization, sensor duty cycle, wireless transmission, battery expenditure, and device-specific hardware constraints. The ongoing collection and analysis of video, audio, and inertial data would entail substantially different energy requirements: facial recording and on-device optical-flow computation are expected to be more resource-demanding than intermittent inertial measurements, while continuous wireless data transfer to an external server could increase energy use and raise privacy and connectivity concerns (64). A feasible future implementation might therefore include task-triggered or scheduled assessments, local feature extraction, quantized or hardware-accelerated inference, and selective activation of the most informative modalities rather than continuous operation of all four streams (65). Such approaches remain directions for future device-specific and real-world validation and should not be interpreted as evidence of current deployment readiness or established at-home clinical use. Future studies should determine whether such approaches can support assessment beyond specialized clinical environments after prospective, device-specific, and real-world validation (61).

From a methodological perspective, the observed heterogeneity in fusion strategy performance highlights the necessity for alignment between integration methods and therapeutic goals. ABF structures, such as dual-attention processes, are beneficial when sensitivity to nuanced multimodal inputs is emphasized, as they can prioritize informative aspects (66). The instability shown in some fusion scenarios (e.g., IF on larger modality sets) underscores the necessity of meticulous validation to prevent bias and overfitting (67). Future development must prioritize adaptive fusion methodologies that can flexibly adjust modality weighting according to context (e.g., home vs. clinical environments) or individual patient profiles, ensuring resilience across varied real-world scenarios (68).

Our study has some limitations. From a technological perspective, pruning reduced selected measures of computational demand within the high-performance GPU environment used for this study. No experiments were performed on wearable, mobile, or edge hardware; consequently, no conclusions can be drawn about device-level feasibility, latency, memory use, energy consumption, or deployment readiness. Furthermore, interpretability remains limited; understanding how each modality contributes to the final classification is crucial for building confidence and trust among physicians and patients. The unimodal architectures were initially developed in our prior research for various PD-related classification tasks other than fall-risk classification. Although task-specific refinements were applied, investigating architectures designed specifically for cross-sectional classification of fall-related vulnerability may be valuable in future research. Additionally, the class distribution should also be considered when interpreting the results. Although the aforementioned imbalance was moderate rather than severe, it may have influenced class-specific estimates, particularly for the faller group. The use of participant-level LOSOCV and f1-score reduced the likelihood of majority-class dominance, but future external studies should assess the framework in larger and more evenly distributed cohorts and report class-specific performance measures.

Additionally, the clinical+ABF analysis was performed using precomputed ABF out-of-fold probability estimates. Because the complete stacking pipeline was not implemented through nested cross-fitting within every outer LOSOCV fold, information from the outer held-out participant may have indirectly influenced probability features used to train the meta-model. Accordingly, the Clinical+ABF results are exploratory and may be optimistic.Moreover, the LLMF analysis has substantial reproducibility limitations. The API call specified the provider alias gpt-5, but an immutable dated model snapshot and returned backend model version were not recorded. Temperature, top-p, maximum output length, and an API-level random seed were not explicitly specified, and the defaults active at the time of inference were used. Each participant–modality configuration was evaluated once; therefore, repeated-run agreement, prompt sensitivity, and the stability of classifications and generated modality weights were not assessed. Provider-side model updates may also affect replication even when the same alias and prompt are used. Furthermore, the generated rationales and optional modality weights were not evaluated for faithfulness and should not be interpreted as mechanistic explanations. Accordingly, LLMF is presented solely as an exploratory proof-of-concept and not as a reproducible benchmark, a state-of-the-art method, or a validated clinical fusion strategy. Finally, the participant-level train/test separation was maintained during model fitting and prediction. However, the final modality-specific pruning ratios were selected by examining aggregate LOSOCV performance across the candidate pruning levels rather than through a fully nested inner model-selection loop. This procedure does not introduce segment-level overlap between training and test participants, but it may produce optimistic selection bias in the reported performance of the chosen pruned configurations.

From a clinical perspective, the study provides an internal participant-level evaluation using data from a two-center cohort, but it does not provide external validation in a geographically, ethnically, or institutionally independent cohort. All participants were recruited from two specialist clinics in Greece, and the cohort size, regional recruitment, eligibility criteria, and controlled assessment setting may restrict generalizability to broader PD populations. Fall risk in PD is influenced by numerous factors not fully encompassed within our analytical framework, including medication effects, comorbidities, and cognitive deficits. Similarly, facial hypomimia should be interpreted as a complementary marker associated with fall-related vulnerability rather than a specific biomarker, because facial dynamics may also reflect overall disease severity, axial dysfunction, medication state, and other motor or non-motor manifestations of PD. To ensure safe and valid participation, we excluded patients with substantial cognitive impairments or comorbidities (e.g., dementia, musculoskeletal limitations) that would hinder completion of physical and vocal tasks. Additionally, all assessments were performed in a controlled laboratory environment to minimize external variability. Although this approach ensured uniform testing conditions, it differs from real-world settings where falls actually occur and may affect the interpretative value of the collected digital signals.

Constraints also stem from the clinical categorization strategy used to stratify fall-related vulnerability. We included a distinct intermediate group of patients exhibiting postural instability during the ON-medication state, but reporting no recent falls, as a clinically meaningful subgroup often overlooked in traditional dichotomous fall vs. no-fall classifications. Their clinical characteristics differed from those of the other groups in several measures; however, the cross-sectional design does not establish that this group represents a transitional stage or that its members will subsequently experience falls. While impaired postural stability and previous falls are among the most consistently reported clinical markers associated with future falls in PD (52), the present study is cross-sectional and, therefore, cannot establish prospective fall prediction. Accordingly, the outcome should be interpreted as a cross-sectional classification of fall-related vulnerability, based on current postural instability and preceding fall history, rather than as an estimate of future fall occurrence. These findings nevertheless support further investigation of multimodal digital approaches for cross-sectional characterization of postural instability and fall-related vulnerability. Postural instability, though, represents only one component of fall vulnerability. Patients without an impaired pull-back test during the ON state may still be at risk due to other fall-related phenomena, such as freezing of gait, troublesome dyskinesia, severe camptocormia, or balance impairment emerging predominantly during the OFF state. Future prospective longitudinal studies should validate these digital markers against subsequent fall occurrence and refine fall-risk stratification by integrating additional clinical dimensions and detailed fall-related information, including recurrent fall frequency.

Finally, the present framework was evaluated using leave-one-subject-out cross-validation within a single cohort and was not externally validated in an independent population. LOSOCV allows for participant-specific separation of training and testing data, but it does not provide a comprehensive evaluation of generalizability across clinical centers, demographics, acquisition methods, or instruments. Therefore, this limitation should be considered when evaluating the reported performance. Moreover, the present study addresses cross-sectional classification of fall-related vulnerability rather than prospective fall prediction. A one-year longitudinal follow-up has been completed for as many of the initial cohort as could be re-evaluated with the same multimodal data-collection technique, and a two-year follow-up is currently underway. These longitudinal data will enable future prospective evaluation of the framework against subsequently documented fall events. The longitudinal data are not part of the current analysis. Independent external validation and prospective evaluation against subsequently documented falls would be required before clinical utility could be assessed.

## 5 Conclusion

This study evaluated modality-specific pruning and multimodal fusion for cross-sectional classification of fall-related vulnerability groups in individuals with PD. Selected pruned configurations retained similar observed classification performance while reducing model size and estimated computational requirements in the study’s experimental environment. Attention-based fusion produced an observed macro-F1 of 91.67% for the face+gait combination. The LLM-assisted face+upper-limb configuration produced an observed macro-F1 of 93.75% in a single recorded exploratory run; because repeated-run stability and exact model-version reproducibility were not established, this value does not demonstrate reproducible superiority or effectiveness as a clinical fusion strategy. Within this cohort, gait and upper-limb signals provided comparatively strong cross-sectional discriminative information, while facial and speech signals contributed additional information in selected fusion configurations. These results support further investigation of facial and speech measures as potential complementary inputs for multimodal assessment. They do not independently validate these measures as clinical biomarkers or establish a causal relationship with falls. The evaluated models combined digitally quantifiable signals associated with motor and non-motor characteristics of PD and discriminated among the prespecified cross-sectional groups within this cohort. Independent validation is required to establish reproducibility, clinical utility, and any prospective association with subsequent falls.

The computational results justify further methodological evaluation but do not establish the feasibility of remote acquisition or resource-constrained implementation. However, the present experiments were conducted in controlled clinical settings, computational performance was not evaluated on representative edge hardware, and the cross-sectional outcome does not support prospective fall prediction or prevention claims. External validation, prospective assessment against subsequent fall events, and testing on intended devices and in real-world environments are therefore required before clinical or home-based use can be considered. Within these limitations, the results support further investigation of multimodal AI as a research approach for cross-sectional characterization and classification of fall-related vulnerability in PD.

## Data Availability

The raw facial-video and voice recordings are not publicly available because they contain potentially identifiable biometric information and cannot be fully anonymized. In accordance with the participant consent and applicable ethical and data-protection requirements, facial videos will not be distributed. De-identified clinical data, derived features, and non-identifiable sensor data may be made available upon reasonable request to the corresponding author/s, subject to approval by the responsible institutions and execution of an appropriate data-use agreement. The analysis code, trained model weights, and complete LLM prompt templates will be made available upon reasonable request.
