# Supplementary material for: Cross-sectional stratification of fall-related vulnerability in Parkinson’s disease through multimodal fusion of speech, gait, facial expression, and upper-limb movement
Source: Front Digit Health. 2026 Sep 23;8:1937343. doi: 10.3389/fdgth.2026.1937343 (PMC13642838; doi:10.3389/fdgth.2026.1937343)
Supplement: Supplementary file 1 [file Datasheet1.pdf]

Supplementary Material

Fig. S1 presents the comprehensive performance metrics (accuracy, precision, recall, and f1-score) for the chosen trimmed unimodal models employed as baselines in the fusion trials. The gait model demonstrated superior performance, achieving a f1-score of 83.06%, with high precision at 93.63% and accuracy at 90.83%, so affirming its efficacy as the principal motor biomarker. The facial model attained a moderate performance with an f1 score of 75.29%, whereas the upper limb modality offered a balanced trade-off between sensitivity and precision (f1 = 69.43%). The speech modality exhibited the lowest performance, achieving a f1-score of 54.16%; however, it was still incorporated into fusion tests due to its potential complementing usefulness. These parameters serve as the foundational reference for evaluating all multimodal fusion techniques.

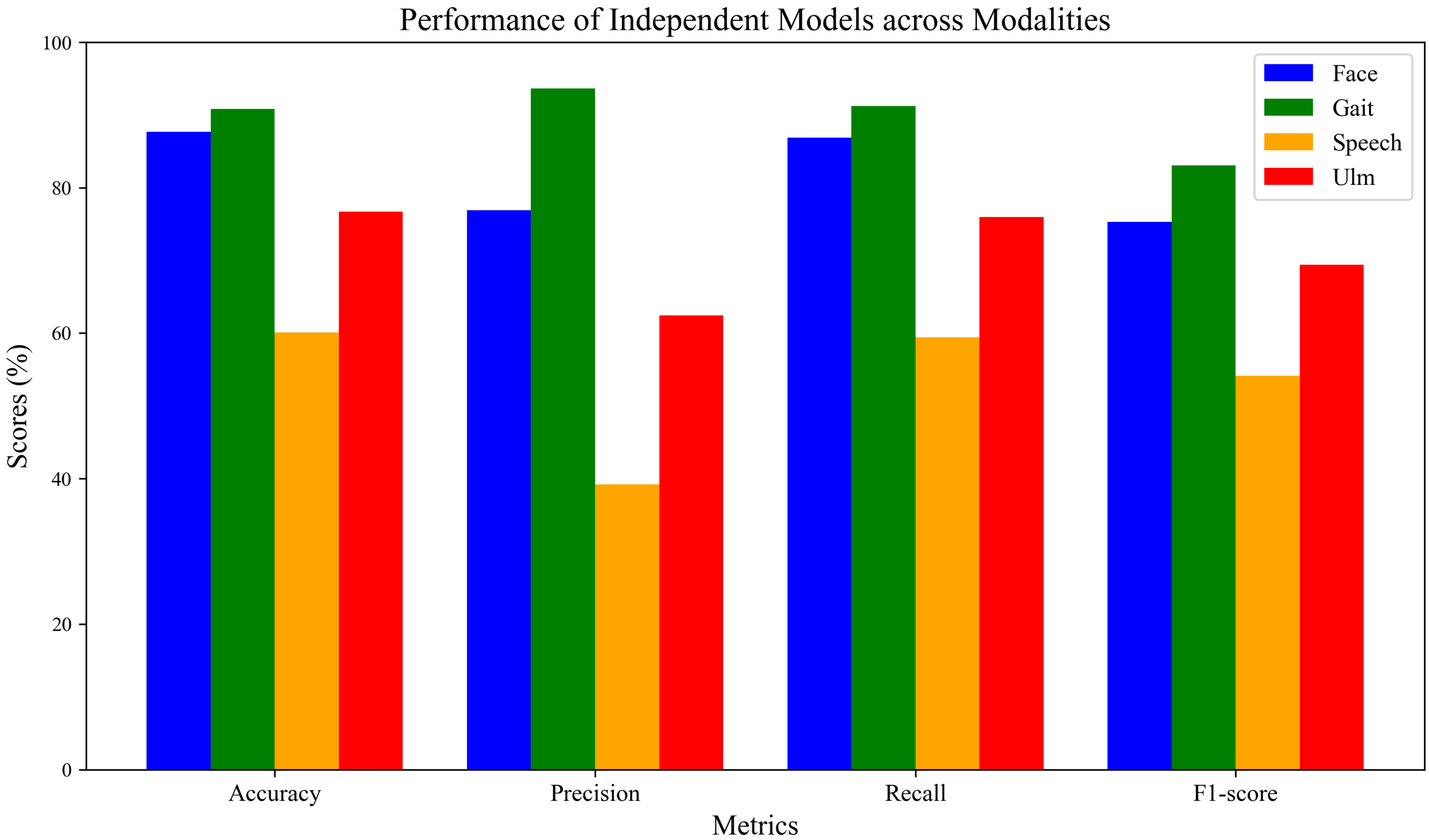

Figure S1. Performance metrics for the four unimodal models after pruning. Gait emerges as the most reliable modality, while speech shows the lowest discriminative capacity. Face and upper limb provide intermediate performance, establishing the basis for subsequent fusion experiments.

Fig. S2 presents the performance of Late Fusion (LF) for all combinations of modalities. LF demonstrated small improvements over unimodal baselines when adding robust modalities, yet exhibited significant limitations when including weaker or noisier signals. The greatest f1-scores were recorded for the face+gait combination (78.62%) and the face+gait+ulm combination (79.46%), both surpassing the unimodal face model and nearing gait performance. The enhancements were chiefly influenced by elevated recall rates (91.10% and 89.72%, respectively), suggesting that LF prioritized sensitivity over precision. The indiscriminate incorporation of speech frequently decreased performance, as evidenced by face+gait+speech

(f1 = 73.42%) and gait+speech (f1 = 64.14%), where precision significantly declined despite robust recall. Likewise, the low precision in ulm+speech (36.06%) led to the most subpar overall result (f1 = 50.16%). The comprehensive four-modality LF fusion attained a f1 score of 74.41%, which is inferior to the unimodal gait model's score of 83.06%, hence underscoring the peril of simplistic averaging when the reliabilities of modalities vary. The results together indicate that although LF can slightly improve performance in certain combinations, its absence of adaptive weighting restricts robustness, especially in diverse modality sets.

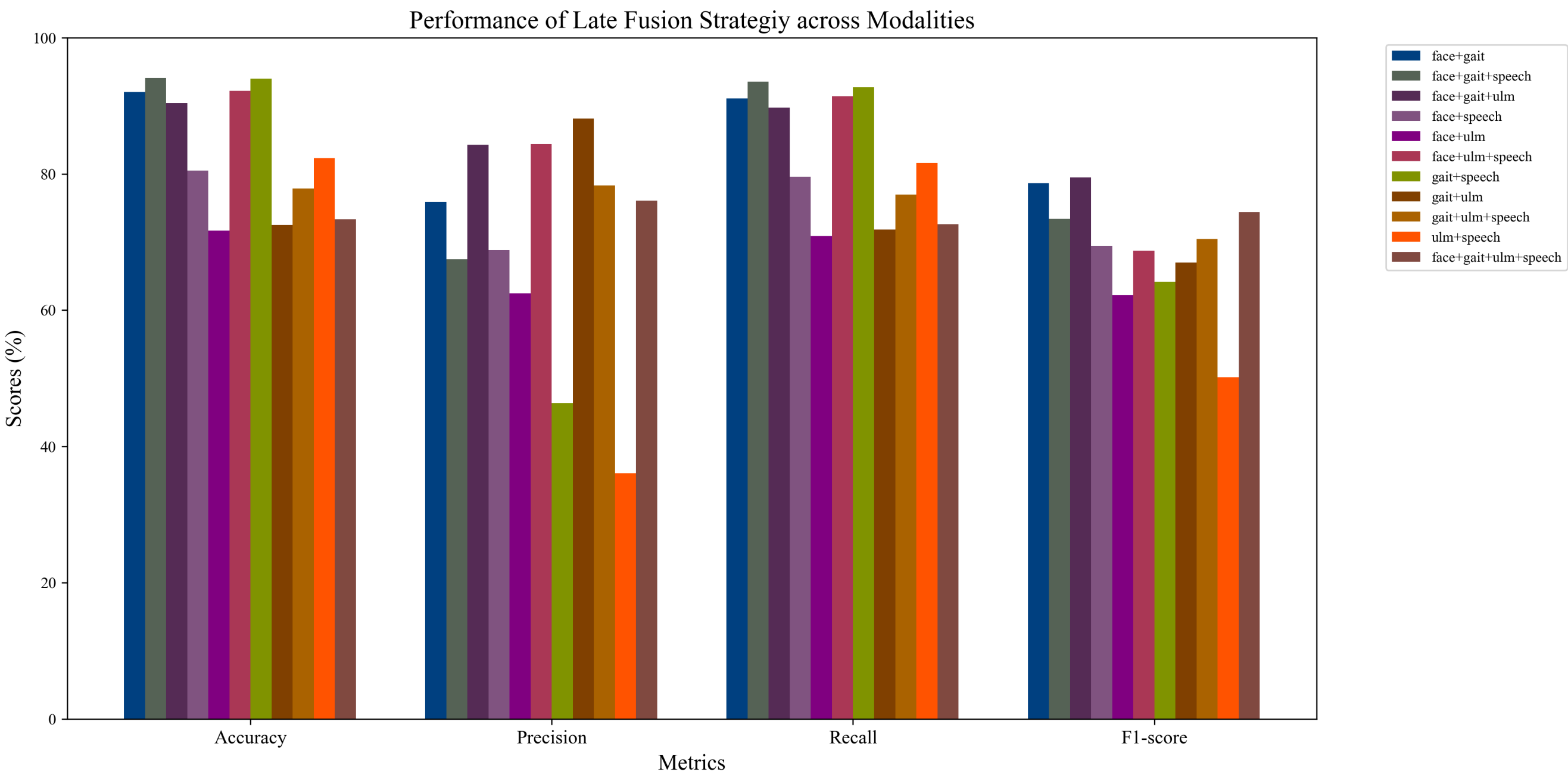

Figure S2. While certain pairs, such as 'face+gait' and 'face+gait+ulm', improved moderately over single-modality baselines, combinations including speech or all four modalities often underperformed relative to the unimodal gait model.

Fig. S3 delineates the efficacy of Intermediate Fusion (IF) across various modality combinations. Overall, IF exhibited inferior performance compared to the pruned unimodal baselines, highlighting the shortcomings of simplistic feature concatenation devoid of adaptive weighting. The optimal configuration was face+gait, attaining a f1 score of 79.64%, with balanced precision at 78.74% and recall at 82.06%. This configuration exhibited a marginal enhancement over the unimodal face model but remained inferior to the gait-only model, which scored 83.06%. Most other combinations demonstrated reduced performance, frequently influenced by slight precision-recall trade-offs. For example, the combination of face, gait, and ulm achieved only 69.11% f1 score, while the combination of gait and speech decreased to 63.16%, indicating that additional modalities frequently increased noise rather than providing complementary signals. The least favorable results were observed for ulm+speech (f1 = 50.96%) and gait+ulm+speech (f1 = 57.46%), both marked by low precision and recall. The four-modality arrangement produced a f1 score of only 59.90%, significantly inferior to the gait baseline. These findings affirm that although IF can achieve modest improvements in specific instances, it encounters difficulties in generalizing across diverse signals, perhaps due to discrepancies in scale and imbalances in modality representations.

Fig. S4 presents the efficacy of Attention-Based Fusion (ABF) across several modality combinations. In contrast to Late Fusion and Intermediate Fusion, ABF consistently exhibited significant improvements across many multimodal contexts by dynamically weighting complimentary signals. The optimal result was

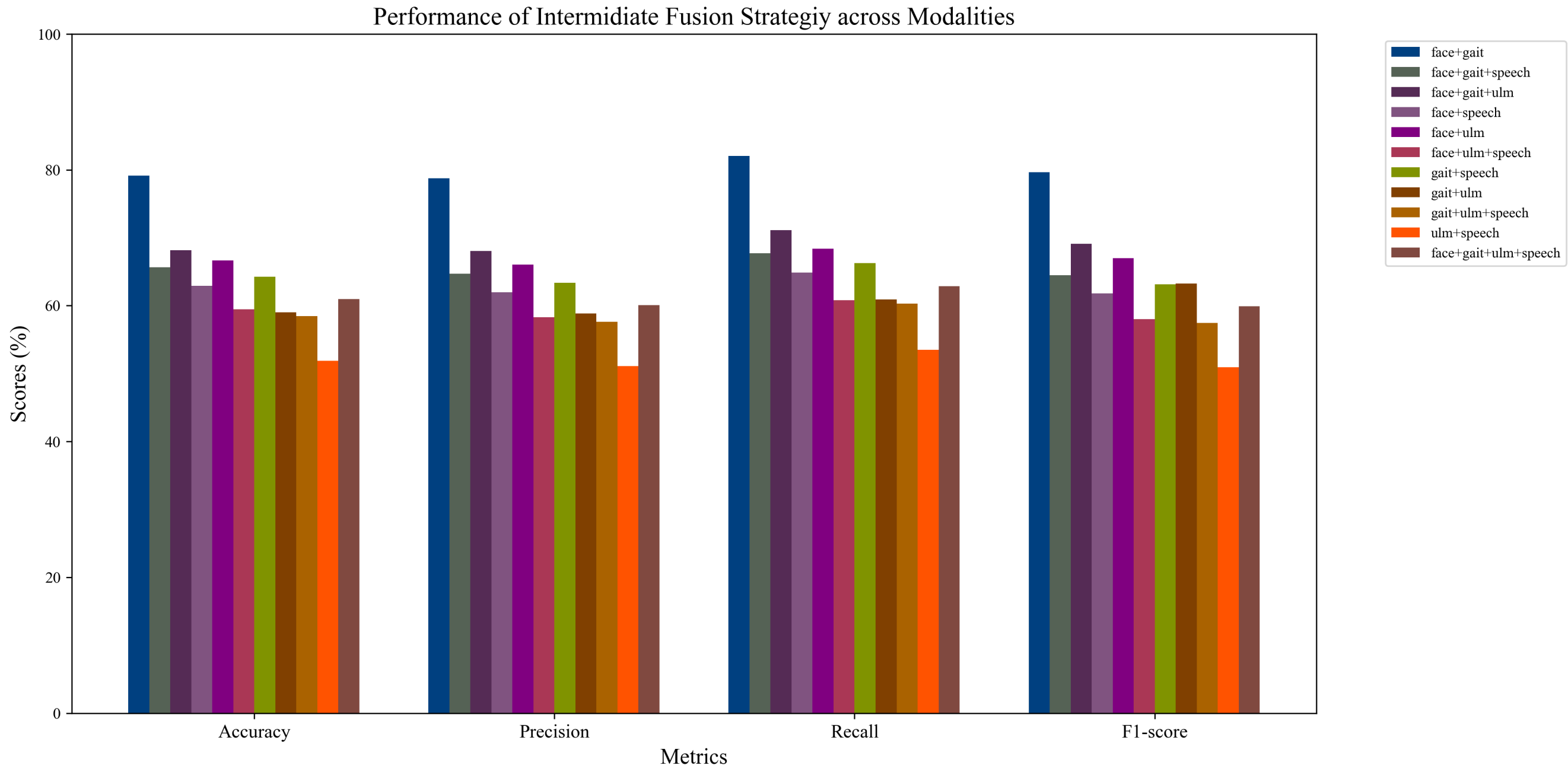

Figure S3. Except for 'face+gait', most configurations underperformed relative to unimodal baselines, with four-modality fusion ranking among the weakest outcomes.

recorded for face+gait, attaining a f1-score of 91.67% with exceptionally high precision (91.67%) and recall (91.67%), surpassing both individual modalities and all alternative fusion procedures in this configuration. The combination of face, gait, and upper-limb dynamics shown strong performance (f1 = 87.34%), underscoring its additional significance. Other beneficial couples comprised face+ulm (f1 = 81.65%) and face+ulm+speech (f1 = 80.93%), both of which enhanced unimodal baselines by optimizing precision and recall. Conversely, combinations that include only speech with gait or facial expressions (face+speech, gait+speech) had inferior performance (f1 = 61–64%), indicating that speech offers diminished predictive value until augmented by more robust modalities. The lowest performance was recorded for the complete four-modality fusion (face+gait+ulm+speech, f1 = 64.81%), where the inclusion of extra signals likely introduced noise. ABF demonstrated superiority over LF and IF, highlighting the advantages of attention mechanisms in selectively prioritizing informative stimuli and suppressing less reliable modalities.

Fig. S5 delineates the performance of Multimodal Transformer Fusion (MTF) across several modality combinations. MTF exhibited significant and equitable results, with the majority of configurations attaining f1-scores near or exceeding the highest unimodal baseline (gait, f1 = 83.06%). The optimal outcome was achieved using all four modalities (facial, gait, upper limb movement, and speech), resulting in an f1 score of 85.02%, accompanied by good accuracy (85.73%), precision (84.10%), and recall (84.85%). Within smaller subsets, face+gait exhibited constant performance (f1 = 83.38%), closely resembling unimodal gait but demonstrating a marginally enhanced equilibrium between precision and recall, whereas face+gait+speech attained 81.27%. Alternative combinations, including gait and speech (f1 = 79.84%) and gait and ulm (f1 = 79.99%), exhibited consistent performance, highlighting the model's capacity to synthesize motor signals. In contrast, combinations characterized by weaker modalities (face+speech, ulm+speech) exhibited significantly inferior performance (f1 = 62–64%), highlighting their restricted discriminative capability when considered independently. MTF demonstrated notable proficiency in managing extensive multimodal

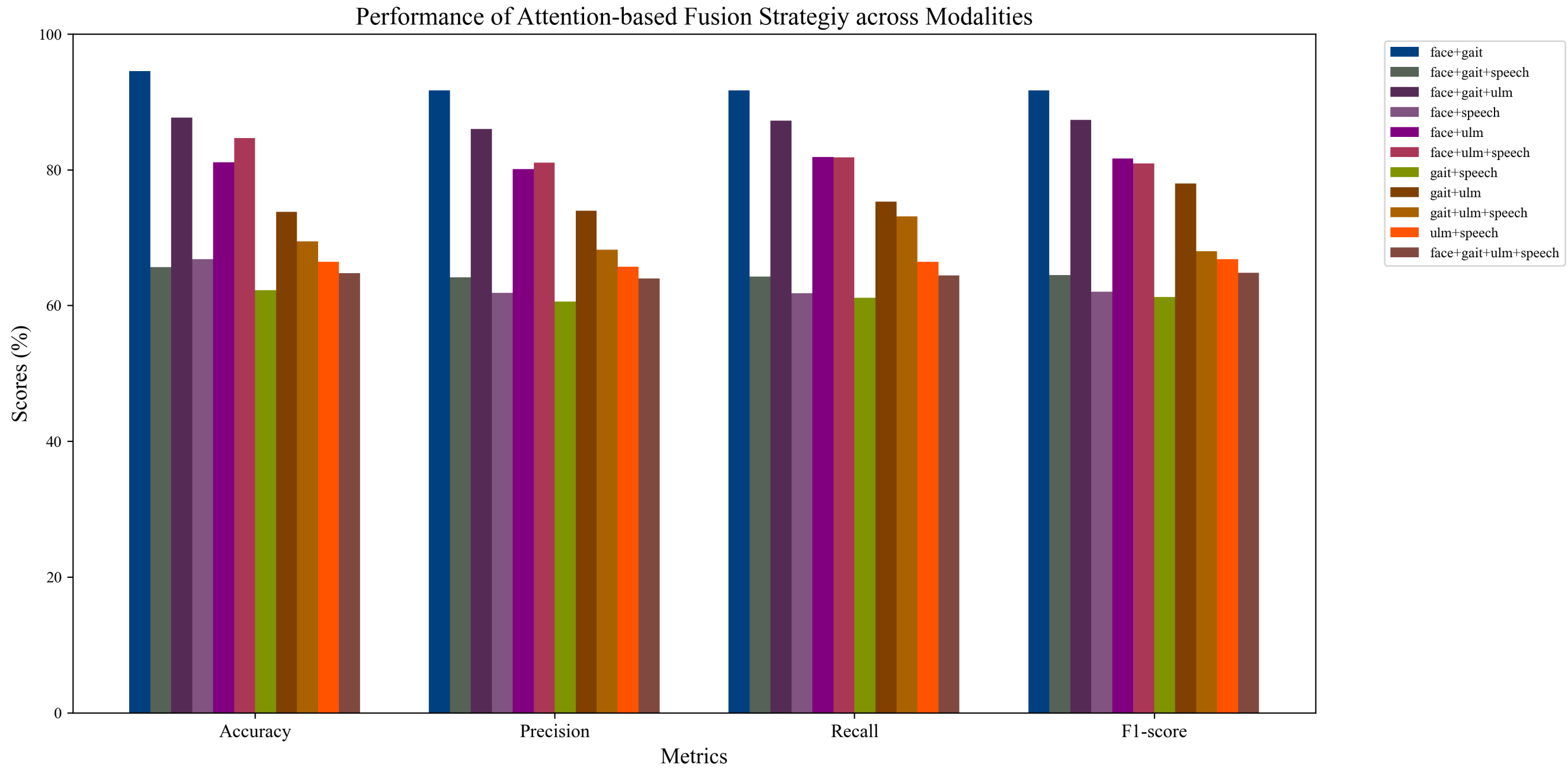

Figure S4. The best outcomes were achieved for 'face+gait' and 'face+gait+ulm', while four-modality fusion substantially underperformed, reflecting the importance of selective modality weighting.

contexts, reaching its zenith with all four modalities, and ensured consistent integration across various modality subsets.

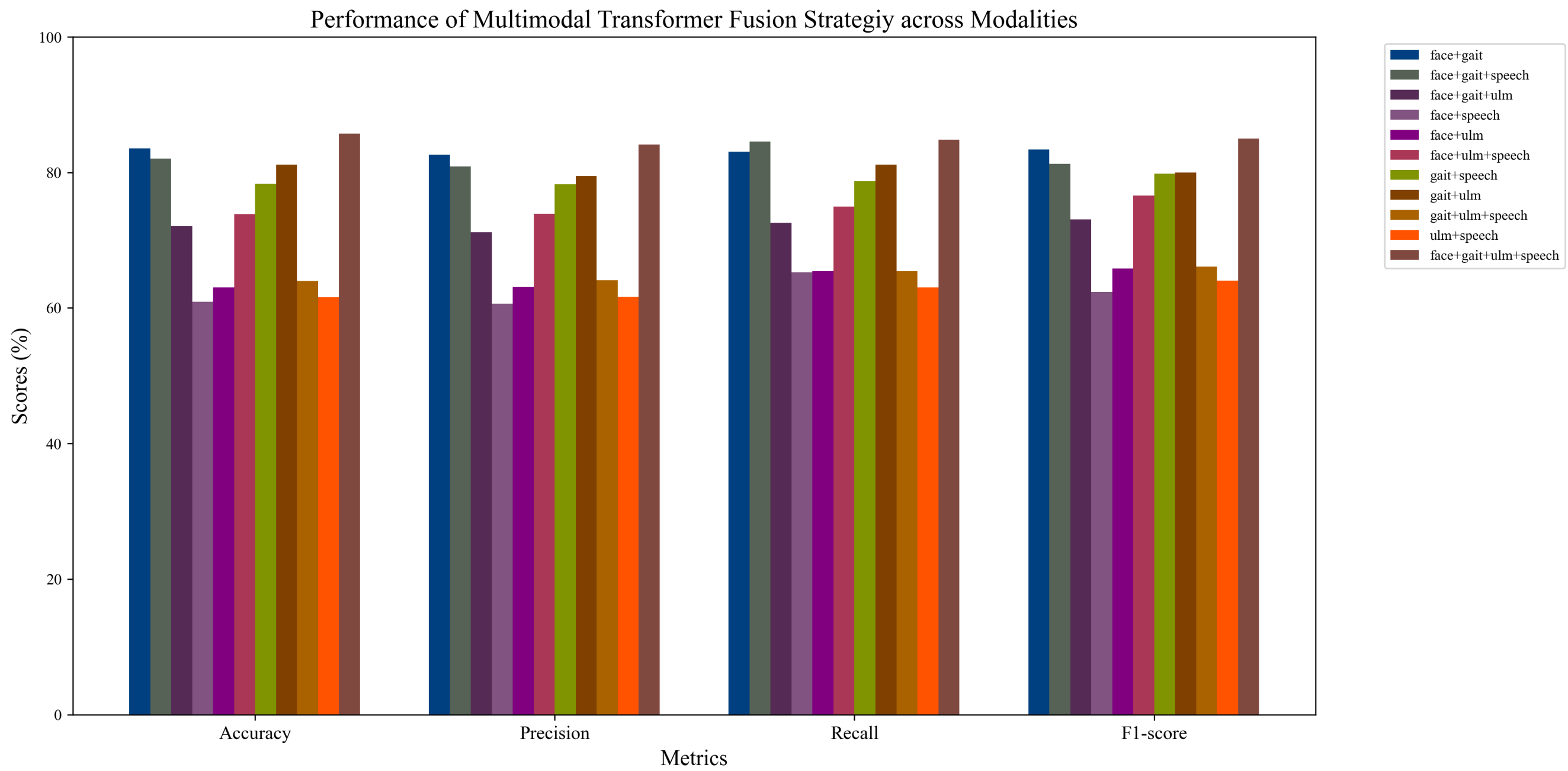

Figure S5. The best performance was achieved with the four-modality fusion, while pairings including gait and upper limb generally delivered stable outcomes, reflecting MTF's strength in balancing multiple information sources.

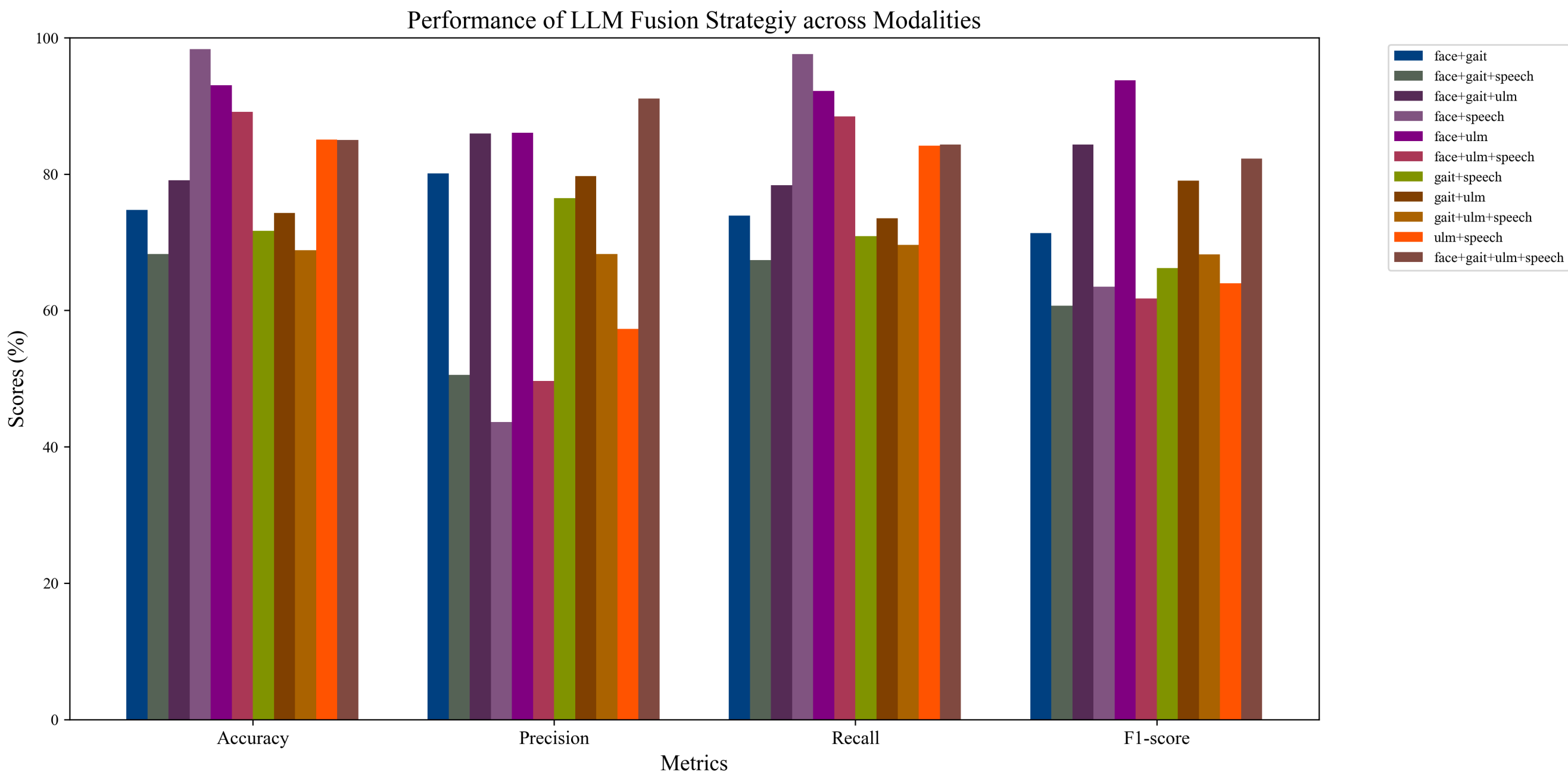

Figure S6. The best-performing configuration was 'face+ulm' (f1 = 93.75%), whereas some other combinations—particularly those including speech—showed weaker performance, reflecting the variability of LLM-driven integration.

Fig. S6 delineates the performance metrics of fusion utilizing Large Language Models (LLMs) across various modality combinations. In contrast to fixed fusion procedures, LLM fusion yielded significantly varying outcomes based on the selected subset of modalities. The exceptional configuration was face+ulm, attaining a f1-score of 93.75%, along with high accuracy (93.01%) and recall (92.18%), establishing it as the superior fusion result among all techniques and combinations. Additional robust outcomes comprised face+gait+ulm (f1 = 84.34%) and the comprehensive four-modality fusion (face+gait+ulm+speech), achieving 82.28%. Motor-centric combinations, such as gait+ulm (f1 = 79.04%), exhibited competitive performance. Nevertheless, specific combinations, especially those incorporating speech, exhibited suboptimal performance—e.g., face+gait+speech (f1 = 60.71%) and face+ulm+speech (f1 = 61.74%)—probably indicating the more erratic and less dependable predictive value of speech within this dataset. LLM fusion shown remarkable adaptability, achieving state-of-the-art performance in certain modality pairs, albeit with increased instability across a wider array of combinations.
